# Genetic Dissection of the Signaling Cascade that Controls Activation of the *Shigella* Type III Secretion System from the Needle Tip

**DOI:** 10.1038/srep27649

**Published:** 2016-06-09

**Authors:** I. Murillo, I. Martinez-Argudo, A. J. Blocker

**Affiliations:** 1 School of Cellular & Molecular Medicine, University of Bristol, BS8 1TD, Bristol, United Kingdom; 2 Área de Genética, Facultad de Ciencias Ambientales y Bioquímica, Universitdad de Castilla-La Mancha, E-45071, Toledo, Spain; 3 Schools of Cellular & Molecular Medicine and Biochemistry, University of Bristol, BS8 1TD, Bristol, United Kingdom

## Abstract

Many Gram-negative bacterial pathogens use type III secretion systems (T3SSs) for virulence. The *Shigella* T3SS consists of a hollow needle, made of MxiH and protruding from the bacterial surface, anchored in both bacterial membranes by multimeric protein rings. Atop the needle lies the tip complex (TC), formed by IpaD and IpaB. Upon physical contact with eukaryotic host cells, T3S is initiated leading to formation of a pore in the eukaryotic cell membrane, which is made of IpaB and IpaC. Through the needle and pore channels, further bacterial proteins are translocated inside the host cell to meditate its invasion. IpaD and the needle are implicated in transduction of the host cell-sensing signal to the T3S apparatus. Furthermore, the sensing-competent TC seems formed of 4 IpaDs topped by 1 IpaB. However, nothing further is known about the activation process. To investigate IpaB’s role during T3SS activation, we isolated secretion-deregulated IpaB mutants using random mutagenesis and a genetic screen. We found *ipaB* point mutations in leading to defects in secretion activation, which sometimes diminished pore insertion and host cell invasion. We also demonstrated IpaB communicates intramolecularly and intermolecularly with IpaD and MxiH within the TC because mutations affecting these interactions impair signal transduction.

Type III secretion systems (T3SSs) are macromolecular structures used by many Gram-negative bacteria. They deliver protein “effectors of virulence” into eukaryotic host cells^1^ to modulate biochemical pathways in favor of the bacterium^2^. We study the T3SS of *Shigella flexneri*, the agent of human bacillary dysentery, focusing on traits conserved in all species, such as physical sensing of host cells.

*Shigella* is an enteropathogen causing ~165 million diarrheal episodes per year worldwide, with a 10% fatality rate for children in the developing world^3^. *Shigella* invades the colonic epithelium. Once inside an epithelial cell, it escapes from the vacuole, replicates within the cytoplasm and disseminates to neighboring cells. *Shigella* is also taken up by macrophages, causing their death by pyroptosis and severe inflammation and by neutrophils, which kill the bacteria, controlling the infection^4^.

The *Shigella* T3SS basal body is anchored in both bacterial membranes and followed by a hollow needle, formed of MxiH, that protrudes from the bacterial surface and acts as the secretion channel^5^,^6^,^7^,^8^. The needle is capped distally by the tip complex (TC), formed of IpaD and IpaB. The TC was proposed as the host cell sensor because without it the bacteria cannot regulate secretion or invade host cells^9^,^10^,^11^,^12^,^13^. MxiH, is a ~9 kDa α-helical hairpin^14^,^15^. It polymerizes into the helical needle using both of its termini^15^,^16^. Single amino acid mutations in needle proteins alter secretion regulation, host cell sensing and TC composition^13^,^17^,^18^. Similar to MxiH, the ~37 kDa IpaD contains a central coiled coil and requires its C-terminus to bind needles^13^,^19^. Point mutations in the upper part of IpaD’s C-terminal helix render the T3SS unresponsive to an artificial inducer of secretion, the small amphipathic dye Congo red (CR^20^) or to host cells^21^. This and its position atop needles indicate it is involved in sensing host cells. IpaD is essential for recruitment of IpaB to TCs^13^. Only one third of the structure of the ~62 kDa hydrophobic IpaB was crystallized, as an ~150 amino acid-long antiparallel coiled coil or alacoil^22^. While IpaB deletion mutants pleiotrophically affect T3SS regulation and host cell invasion^23^,^24^, a direct role for IpaB in host cell sensing remains uninvestigated.

While others suggest IpaB is added atop needles after exposure to the bile salt deoxycholate (DOC^25^,^26^,^27^), we find IpaB in TCs without DOC addition^12^,^13^. Three-dimensional reconstruction of the resting *Shigella* TC using electron microscopy demonstrates a TC subset contains 4 IpaDs and 1 IpaB^12^. The remainder of TCs at the bacterial surface contain 5 IpaDs, as also reported by other groups^28^,^29^. At the helical needle tip, the 11 MxiH protofilaments generate 5 subunit-binding sites. Four out of the five potential insertion sites are equivalent but the lowest is unique because it is bound by two non-continuously rising subunits^11^. Five IpaDs may initially polymerize at the needle tip, with IpaB then replacing an IpaD at the unique site and protruding above them^12^. However, it is unclear which TCs are functional for sensing.

IpaB binds cholesterol and CD44 in the host cell plasma membrane^30^,^31^. Its hydrophobic regions become inserted into the host membrane, where it becomes part of the effector translocation pore (translocon), along side the hydrophobic IpaC^6^. IpaB is also involved in T3SS regulation, through transcriptional regulation of some effectors. Indeed, it first sequesters then releases its intrabacterial chaperone, IpgC, upon its own secretion^32^,^33^. Free IpgC binds MxiE, functioning as transcriptional co-activator of later acting effectors^34^,^35^. Finally, IpaB is involved in invasion vacuole lysis^36^,^37^ and binds caspase-1 to activate macrophage pyroptosis^38^.

IpaB contains a bipartite chaperone-binding site (residues 16–72^39^; Fig. 1A). Its N-terminal alacoil region is located between residues 74 and 224^22^ and its IpaC binding domain at residues 367–458^40^. Between these, IpaB carries an amphipathic α-helix (residues 240–280) and a hydrophobic domain (residues 310–430) containing two predicted transmembrane helices (residues 313–346 and 400–423^41^). IpaB is also predicted a C-terminal coiled-coil forming α-helix (residues 530–580). Its extreme C-terminus is required for needle binding and secretion regulation^23^. This would place the IpaB coiled-coil and C-terminal globular domains in a topologically equivalent position to those of IpaD atop TCs, optimally positioning its hydrophobic regions to interact with host cell membranes^19^,^23^,^24^.

Prior to contact with cells, TC proteins not already in the tip are cytoplasmically stored^32^,^42^. Upon host cell sensing, the TC transmits an unknown signal via the needle into the cytoplasm, activating secretion^17^,^43^. Given its situation in the TC^12^ and its essential role in host cell membrane penetration and translocon formation^6^, IpaB is likely the host-cell sensor, while IpaD is the first element of the signal transduction cascade^13^. Activation triggers the release of IpaC, forming the translocon in the host membrane along with IpaB atop the TC^6^,^23^,^24^,^43^, while IpaD acts as an adaptor between needle and pore^13^,^44^. Translocon insertion triggers a second signal that travels down the needle to induce effector secretion^17^,^43^.

To summarize, IpaD, IpaB and IpaC are dispensable for secretion, but essential for effector injection in a manner that is still not understood. Upstream of this event, IpaD and IpaB are essential for regulation of secretion^13^,^45^,^46^. Cumulative evidence shows the TC is involved in host cell sensing^12^,^13^,^23^,^24^,^43^ but this infection-initiating event remains mechanistically mysterious. Physical interactions between IpaB, IpaD and the needle tip are central to this process^12^,^21^,^23^,^24^,^29^. But, how remains unexplored.

To test whether IpaB is directly involved in host sensing, we isolated *ipaB* mutants unresponsive to activation signals. We used a genetic screen for mutants insensitive to induction by CR^21^. We identified seven *ipaB* single point mutations preventing CR-mediated secretion activation. All but one localized to IpaB’s alacoil. Although they all showed normal TC composition some were also impaired in host cell interactions. By combining *in cis* the newly isolated *ipaB* mutations with a short C-terminal deletion^23^, we uncovered crosstalk between different IpaB regions. Expression of either type of *ipaB* mutations *in trans* both with *ipaD* mutants with similar phenotypes and with a constitutively secreting needle mutant^17^,^21^ also uncovered epistasis. Overall, we determined which regions of IpaB communicate with which in itself, IpaD and MxiH in TCs, and that failures in these interactions impair signal transduction. Hence, conformational changes during IpaB membrane-insertion may initiate T3SS activation.

## Results

Prior to host cell contact, only the Ipa proteins and another early effector are synthesized (i.e., IpaA^47^, IpaB, IpaC, IpaD and IpgD^48^), with ~5% of these being released slowly via the apparatus. This is termed “leakage”. “Induction” is the burst of Ipa protein secretion upon host cell contact^45^. This may be mimicked by CR addition^20^, when secretion of 50% of Ipas and IpgD is detected in 15 min. Deregulated leakage, termed “constitutive secretion” involves high levels of secretion of Ipa proteins, IpgD and late effectors. Some *mxiH* mutants lead to “slow” constitutive secretion, detectable in hours^17^. Deletion of *ipaD* or *ipaB* leads to “fast” constitutive secretion, detectable in minutes, and to CR unresponsiveness^13^. The physiological relevance of these secretion states is unclear^12^ but they are useful experimental tools and understanding their differences will help follow our results.

### IpaB must be secreted to exert its regulatory function

**Figure 1:**
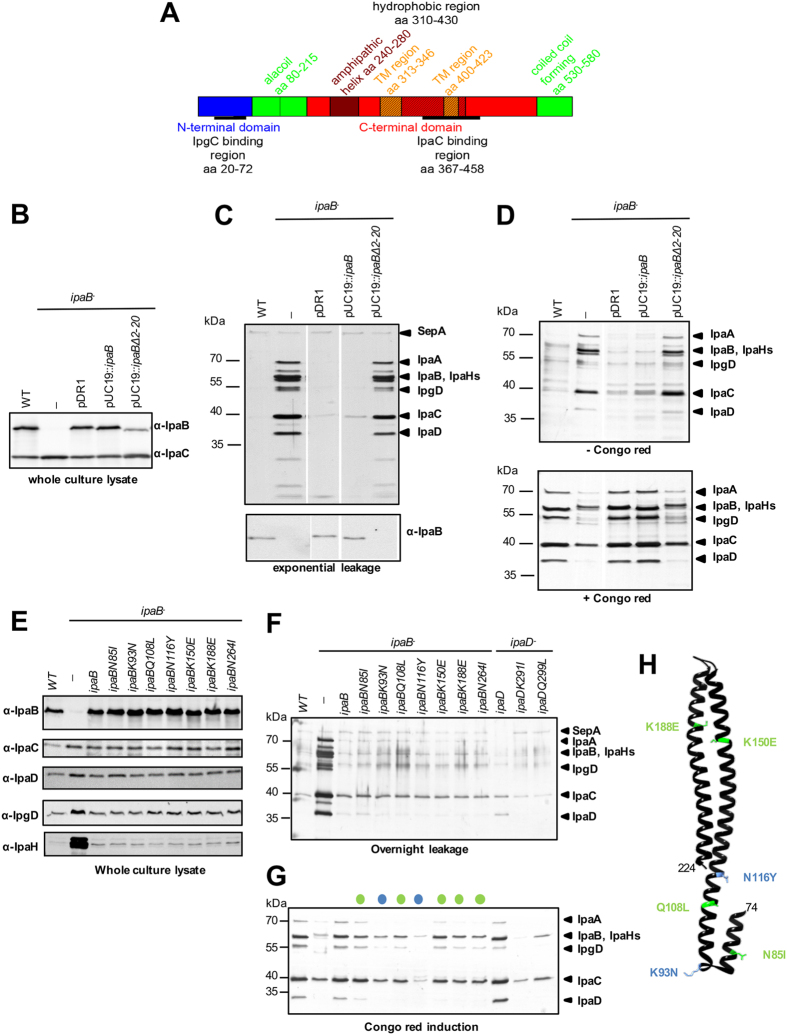
Characterization *ipaB*Δ2-20 and ipaB* mutants. (**A**) Linear representation of IpaB secondary structure predictions and domain assignments. (**B**) Expression levels of IpaB and IpaC in cultures of *S. flexneri* wild-type (WT), *ipaB*^−^, and pDR1 and pUC19::*ipaB* (complementation plasmids) and *ipaB*Δ2-20 in *ipaB*^−^. (**C**) Exponential culture supernatants from strains in B were Silver stained (*top*) or blotted against IpaB (*bottom*). (**D**) Protein secretion in response to absence (*top*) or presence (*bottom*) of CR, analyzed by Silver staining. (**E**) Expression of indicated antigens in cultures of WT, *ipaB*^−^, complemented strain (*ipaB*^−^/*ipaB*) and ipaB* mutants in *ipaB*^−^. (**F**) Overnight leakage into the culture supernatant of ipaB* and ipaD* 21 mutants in *ipaB*^−^ and *ipaD*^−^, respectively, analyzed by Silver staining. (**G**) Protein secretion of strains in (**F**) in response to CR, analyzed by Silver staining. Colored dots represent degrees of CR induction reduction: strong (*blue*) and mild (*green*). Results shown are representative of at least two independent experiments. (**H**) Location of 6 out of 7 of the IpaB* mutants within the alacoil structure of IpaB (3U0C^22^). Native amino acids that were mutated are shown as stick models.

To resolve whether IpaB must be exposed on the cell surface to assemble functional TCs, we made an *ipaB* mutant lacking its first 20 amino acids, predicted to contain the secretion signal^49^. *ipaB∆2-20* (Table 1) expressed IpaB at 35% of the level of WT (Fig. 1B), presumably because it binds its chaperone with reduced efficiency. We found that expression of below 20% wild-type IpaB levels in *ipaB*^−^ leads to maximal fast constitutive secretion while 50% of normal IpaB levels greatly reduces it (Fig. S1). IpaB∆2-20 is not secreted (Fig. 1C, *bottom*) and *ipaB∆2-20* displays fast constitutive secretion and CR unresponsiveness, as in *ipaB*^−^ (Fig. 1C, *top* and 1D). Hence, *ipaB∆2-20* causes maximal constitutive secretion because it cannot be secreted, leaving the TC immature and hence dysfunctional.

### All but one *ipaB* mutation unresponsive to CR localize to the alacoil

To assess IpaB’s involvement in sensing the activation signal, we searched for *ipaB* mutants blocked in secretion activation. For this, we screened a library of *ipaB* mutants based on their color on plates containing CR. Wild-type *Shigella* are orange on CR plates^50^. This may reflect secretion of early and late effectors in response to CR. Bacteria lacking functioning T3SSs are white^51^ and those lacking *ipaD* or *ipaB* are red^46^, presumably because they secrete more late effectors.

A library of random *ipaB* mutants was transformed into *ipaB*^−^. Around 1.45 × 10^6^ transformants were screened, 241 white clones isolated and 35 white mutants confirmed by sequencing (Materials and Methods; Table 1). Some mutations appeared more than once and occasionally more than one mutation was found in *ipaB*. Individual mutations were separated to identify which was responsible for loss of CR-sensing capacity (Table 1). Only single point mutations causing the white phenotype, hereafter termed ipaB^*^, were further investigated.

No ipaB^*^ mutant was altered in its expression (Fig. 1E, *top*), ability to store and leak others Ipas and IpgD (Figs 1E, *middle panels* and 3F) or to repress expression of the late effector IpaH (Fig. 1E, *bottom*). However, these mutants showed degrees of reduced sensitivity to CR-induction that, for some, was similar to that seen for previously characterized CR-insensitive *ipaD* mutants^21^ (Fig. 1G; quantified in Fig. S2). All but one mutation (*ipaBN264I*) localized to IpaB’s alacoil (Fig. 1H). In total, 48% of the independently identified 43 mutations sequenced localized to the alacoil (amino acids 74–224), which encompasses only 26% of the protein length. This suggests the region is key to secretion initiation. Mutants had strong (*ipaBK93N* and *ipaBN116Y*) or mild defects in secretion (*ipaBN85I, ipaBQ108L, ipaBK150E, ipaBK188E* and *ipaBN264I*) in spite of similar expression levels (Fig. 1E, *top*). Therefore, their phenotype is due to a direct effect of the mutations on IpaB function.

### The IpaB* mutants are secreted in a constitutive secretor background

To assess if the ipaB* mutations impaired secretion of IpaB, and hence perhaps secretion of the other Ipa/Ipg proteins, we used constitutive secretor *ipaD*^−^ *ipaB*^−^ 21. The ipaB* mutants were transformed into this background and their secretion profile analyzed by Western blot. IpaB* mutants were expressed and secreted at the same levels as wild-type IpaB (Fig. S3A), indicating the newly isolated mutations do not affect IpaB’s ability to be secreted.

### Most IpaB* mutants form TCs with normal composition

**Figure 2:**
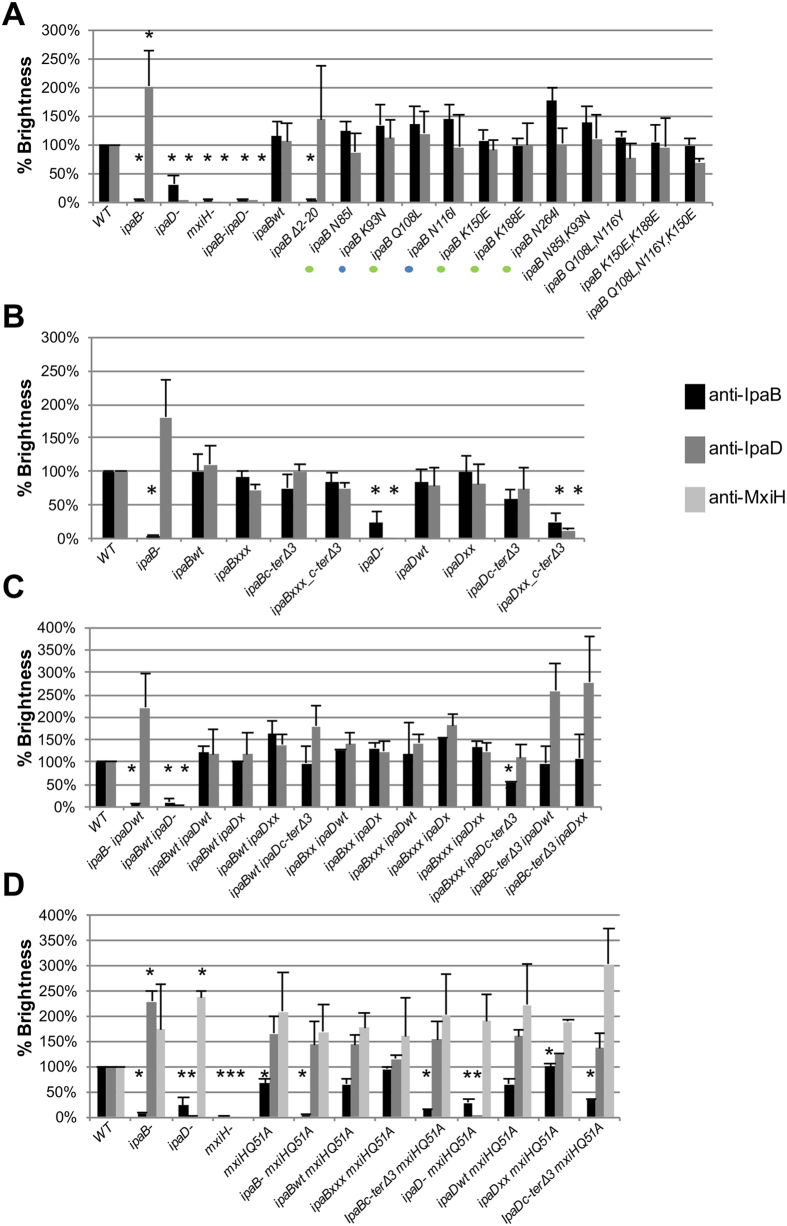
Analysis of IpaB, IpaD and MxiH at the *Shigella* surface by FACS. Strains were analyzed using antibodies against IpaB, IpaD and MxiH. (**A**) Percent brightness of *ipaB*^−^, *ipaD*^−^*, mxiH*^−^*, ipaB*^−^ *ipaD*^−^, complemented strain *(ipaBwt)* and *ipaB* mutants in *ipaB*^−^. Colored dots represent degrees of CR induction reduction, as in Fig. 1G. (**B**) *In cis* combination of *ipaB* and *ipaD* mutations in *ipaB*^−^ and *ipaD*^−^, respectively. For *ipaB* mutant strains, results shown are representative of two independent experiments. (**C**) *In trans* combination of *ipaB* and *ipaD* mutations in *ipaB*^−^ *ipaD*^−^. Mutants were compared to *ipaBwt ipaDwt*. (**D**) Combination of *ipaB* or *ipaD* and *mxiHQ51A* mutations in *ipaB*^−^ *mxiH*^−^ or *ipaD*^−^ *mxiH*^−^, respectively. Mutants were compared against strains *ipaBwt mxiHQ51A* or *ipaDwt mxiHQ51A*. Single mutants were compared to *WT*. Values derive from ≥2 × 10^5^ events per sample. Values were normalized against WT after subtraction of background and compared against corresponding complemented strains. Data presented are the arithmetic mean of the geometric means from, unless otherwise stated, at least three independent experiments. Standard deviations of the means are indicated with bars. Asterisks indicate statistically significant differences (*p* ≤ 0.02) between samples, calculated with Student’s *t* test (type 3 (**A,C**), type 2 (**B,D**)) after ANOVA.

We next used fluorescence-activated cell sorting (FACS) to assess the overall composition of TCs of individual ipaB* mutant cells by immunolabeling the surface of fixed bacteria. The specificity of the antibodies was verified by immunofluorescence (Fig. S4). As negative controls we used *mxiH*^−^, *ipaB*^−^ and *ipaD*^−^, which cannot form needles and/or TCs^7^. As expected *ipaB*^−^, *ipaD*^−^, *ipaB*^−^ *ipaD*^−^ and *mxiH*^−^ showed no/very reduced IpaB staining. Six out of seven ipaB* mutants showed normal TC composition. *ipaBN264I* showed a slightly higher average amount of IpaB at the bacterial surface (statistically significant at p = 0.05 but not at p = 0.02) although it did not show any change in the amount of IpaD (Fig. 2A). Thus, the number of needles and TCs it carries is same as in *WT*. Therefore, *ipaBN264I* could affect the accessibility of this mutant IpaB to the antibodies used for FACS, perhaps reflecting its altered conformation in TCs. The data above indicate all IpaB* mutants localize to TCs. Therefore, isolation of CR-insensitive *ipaB* point mutants suggests IpaB is directly involved in mediating CR responsiveness.

### Some ipaB* mutants form translocons poorly

**Figure 3:**
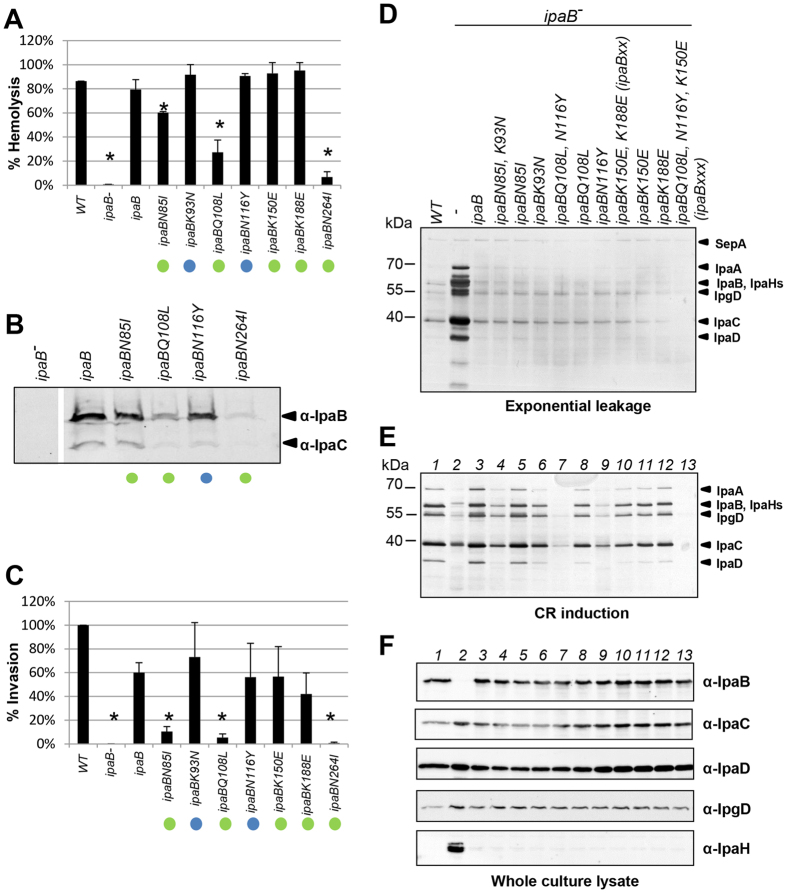
Analysis of ipaB* single mutants host-cell interaction properties and of secretion phenotypes of combinations of ipaB* mutants. (**A**) Hemolytic activity of ipaB* mutants. Values were normalized against those obtained with detergent addition after subtraction of background from RBCs incubated in PBS. Data are averages from ≥3 experiments performed in triplicate; error bars indicate standard deviations. Asterisks indicate statistically significant differences (p < 0.05) between samples compared against the complemented strain, calculated with Student’s *t* Test (type 3) after ANOVA. (**B**) Association of IpaB and IpaC from selected ipaB* mutants with RBC membranes. Samples were normalized by protein concentration. Figure is representative of three experiments. (**C**) HeLa cell invasion by ipaB* mutants. Experiments were normalized against WT. Data represent the mean of ≥3 independent experiments performed in triplicate; error bars indicate standard deviations. Asterisks indicate statistically significant difference (p ≤ 0.05), assessed as above. Colored dots represent degrees of CR induction reduction, as in Fig. 1G. (**D**) Overnight leakage of WT, *ipaB*^−^, *ipaB*^−^/*ipaB* and ipaB* mutants in *ipaB*^−^. (**E**) Their protein secretion in response to CR. (**F**) Protein expression levels in their cultures. Samples were Silver stained (**D,E**) or blotted with indicated antibodies (**F**). Results shown are representative of ≥2 independent experiments.

As IpaB’s membrane-insertion is necessary for epithelial cell invasion, we studied the effect of the ipaB* mutations on pore formation using contact hemolysis. Indeed, *Shigella* lyses Red Blood Cells (RBCs) upon physical contact with them^6^, due to membrane insertion of IpaB and IpaC, which form a pore within RBC membranes^6^. *ipaB*^−/+^ and *WT* showed 85–80% of detergent-mediated hemoglobin release, which is set as 100% hemolysis in this assay (Fig. 3A). Some mutants had normal hemolytic capacity (*ipaBK93N, ipaBN116Y, ipaBK150E, ipaBK188E*), others showed only 60–30% of total hemolysis (*ipaBN85I* and *ipaBQ108L*), while *ipaBN264I* had none. Mutants with the strongest unresponsiveness to CR (*ipaBK93N, ipaBN116Y*) displayed hemolytic activities similar to WT. Thus, the ability to respond to CR is genetically dissociable from the ability to perform hemolysis.

Was the decrease in hemolytic activity of some ipaB* mutants due to a problem in membrane-insertion of mutant IpaB? For those mutants with reduced hemolytic activity, we examined the composition of the lysed RBC membranes isolated by floatation in a sucrose density gradient. We also studied *ipaBN116Y* as the mutant with the greatest reduction in CR induction. Since functional IpaB is a prerequisite for membrane insertion of IpaC^6^, no IpaB and little IpaC were detected in RBCs exposed to *ipaB*^−^ (Fig. 3B). In the membrane fractions of RBCs incubated with *ipaBN85I* and *ipaBN116Y*, the amount of IpaB was less (48% ± 16 and 58% ± 25 reduction relative to *ipaB*^−/+^, respectively; Fig. S3B). For *ipaBQ108L*, the amount of IpaB detected was even less (75% ± 4 reduction). Mutant *ipaBN264I* showed little IpaB (94% ± 2 reduction) associated with RBC membranes. All mutants showed proportional reductions in IpaC insertion (Figs 3B and S3B). N264 is found in the amphipathic α-helix of IpaB (Fig. 1A), which is important for interaction with lipids vesicles^52^. Its polar side chain seems required for interaction with lipid bilayers.

### There is little correlation between the ipaB* mutants’ abilities to sense CR and invade host cells

To evaluate ability of the mutants to invade epithelial cells, we measured protection from Gentamicin upon entry into HeLa cells, since this antibiotic cannot penetrate host cells. *ipaBN85I* and *ipaBQ108L* did not complement *ipaB*^−^ for cell invasion efficiently and *ipaBN264I* failed to restore invasion (Fig. 3C). Thus, there is fairly good correlation between the capacities of the mutants to perform hemolysis and invasion. In contrast, some showed strong defects in CR responsiveness but were unaffected in hemolysis and invasion (*ipaBK93N* and *ipaBN116Y*). This indicates that their capacities to sense CR and host cells are dissociable genetically.

### Combinations of ipaB* mutations enhance CR unresponsiveness

To assess whether the IpaB alacoil folds *in vivo* as it does in the crystal structure, we combined mutations within amino acids nearby in the structure (Fig. 1H) to investigate whether their combination produces stronger phenotypes.

While all combinations of mutants formed had normal TC composition (Fig. 2A), *ipaBN85I, K93N* and *ipaBK150E, K188E* (termed *ipaBxx* from now on) showed slightly enhanced inability to respond to CR relative to each single mutant whereas for *ipaBQ108L, N116Y* the enhancement was greater (Fig. 3D,E). *ipaBQ108L, N116Y, K150E* (hereafter termed *ipaBxxx*) showed a slight, if reproducible, reduction in leakage and complete uninducibility. Since in *ipaBxxx* the altered amino acids are far apart, the stronger phenotypes are likely not due to the amino acids co-assessed being close in the structure. However, that some IpaB* mutations enhance others suggests they produce incremental, structurally-related effects.

Given the lack of correlation between CR-sensitivity and host cell sensing ability in ipaB* mutants, is there any correlation between these phenomena for IpaB? To answer this, we tested the invasive capacity of *ipaBxx* and *ipaBxxx* (Fig. S5). Unsurprisingly given that *ipaBQ108L* is non-invasive, *ipaBxxx* is also. More informatively, *ipaBxx* is also non-invasive, when both *ipaBK150E* and *ipaBK188E* are *WT*-like for invasion. Thus, several mutations in IpaB’s alacoil region, especially when combined, do affect host cell sensing. This suggests the alacoil is involved in transmission of both the CR and host-cell sensing signals. However, it seems less sensitive to the latter.

### There is intramolecular crosstalk between IpaB regions

**Figure 4:**
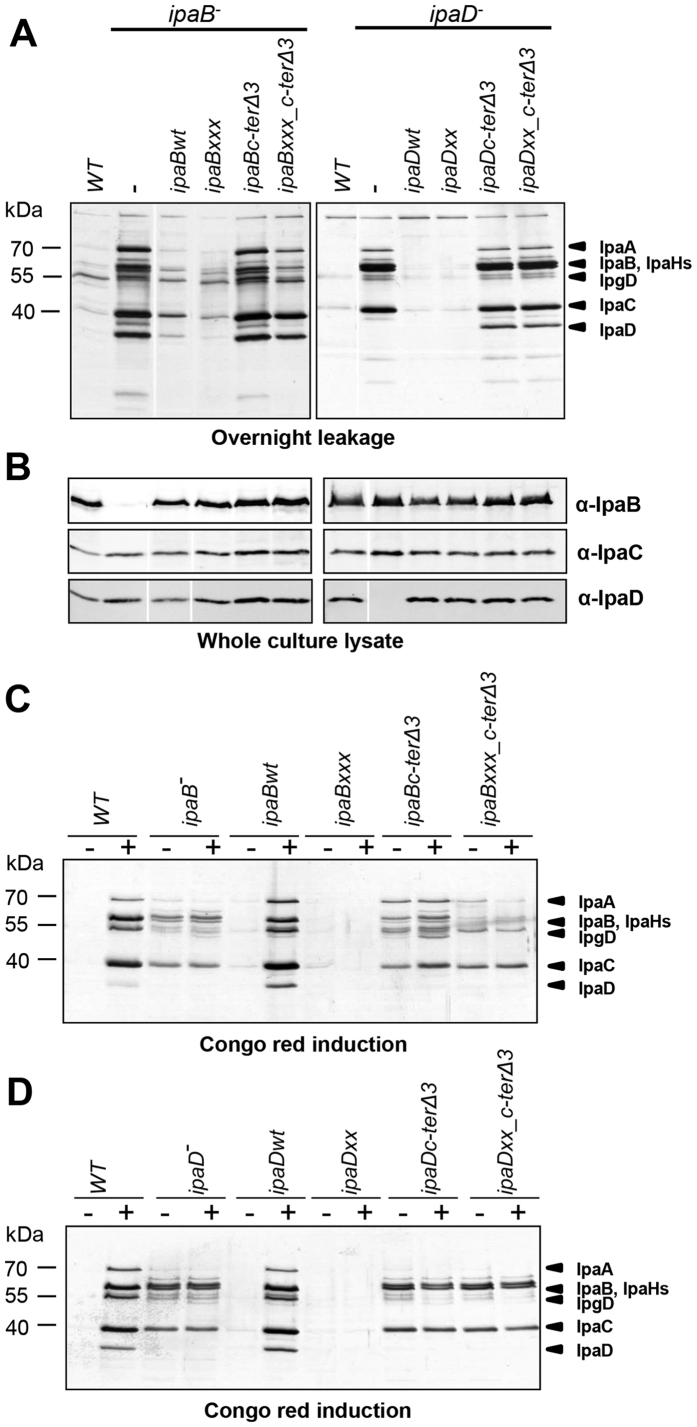
Characterization of *in cis* combined *ipaB* or *ipaD* mutants. Combinations of ipaB* mutations and an IpaB C-terminal truncation in *ipaB*^−^ (*left*) and of similar IpaD mutations in *ipaD*^−^ (*right*) were studied. (**A**) Overnight leakage of indicated strains, analyzed by Silver staining. (**B**) Expression levels of the translocators in total cultures, analyzed by blotting. The images shown are from the same experiment but irrelevant intervening lanes were removed. (**C,D**) Proteins secreted in response to CR, analyzed by Silver staining. Results shown are representative of at least two independent experiments.

Could we alter the combined ipaB* mutant’s secretion phenotypes? In *ipaBc-ter*Δ*3*, IpaB is expressed lacking its last three C-terminal amino acids, making the T3SS constitutively active and weakly inducible by CR^23^. Hence, we examined if the secretion patterns of *ipaBxxx* are altered when expressed in an *ipaBc-ter*Δ*3* background. This combined mutant showed a new, intermediate phenotype: reduced constitutive secretion and reduced CR-induction relative to *ipaBc-ter*Δ*3* (Fig. 4A,B, *left*, C). This indicates epistasis between these sets of mutations, suggesting intramolecular crosstalk between these IpaB domains, where the alacoil region acts upstream of the C-terminus.

### Dual modification of IpaD’s C-terminus leads to loss of needle tip binding

We previously isolated *ipaD* mutants with decreased CR responsiveness^21^ and others characterized *ipaD*Δ*330-332*, termed here *ipaDc-ter*Δ*3*, as a constitutive secretor^29^. To assess the effect of combinations of these mutations on IpaD, the other TC component, we combined *ipaDN186I, K291I* (*ipaDxx*^21^), an ipaD* mutant with strongly reduced secretion, *in cis* with *ipaDc-ter*Δ*3.* Contrary to what happened in *ipaBxxx_c-ter*Δ*3, ipaDxx* had no effect on *ipaDc-ter*Δ*3* (Fig. 4A,B, *right*, D): *ipaDxx_c-ter*Δ*3* behaved as a constitutive secretor. However, all mutants expressed similar Ipa/Ipg levels (Fig. 4B), ruling out deleterious decreases in protein expression.

To understand these contrasting results, we assessed the TC composition of these mutants by FACS (Fig. 2B). *ipaBxxx, ipaBc-ter*Δ*3* and *ipaBxxx_c-ter*Δ*3* have the same tip composition as *ipaB*^−/+^. However, *ipaDxx_c-ter*Δ*3* displays a strong decrease in IpaD (and hence IpaB) when compared with *ipaDxx* and *ipaDc-ter*Δ*3*. Thus, IpaDxx_c-terΔ3 can not bind the needle tip. This may be due to localization of N186 and K291 near or within the C-terminal helix of IpaD. As TC composition is wild type-like for *ipaDxx* and *ipaDc-ter∆3* mutants, this also suggests they are affected in signaling from the needle tip and not a downstream step.

### *In trans* combination of CR-insensitive *ipaB* or *ipaD* mutants and C-terminal deletions generates new phenotypes

To assess whether IpaB and IpaD communicate within the TC, we constructed a series of *ipaB* and *ipaD* mutants *in trans,* which we transformed into *ipaB*^−^ *ipaD*^−^. To reveal phenotypic changes, we combined mutants showing mild phenotypes, *ipaBxx* and *ipaDK291E* (*ipaDx*) with others displaying strongly impaired secretion, *ipaBxxx* and *ipaDxx*. We also combined these mutants with mutants exhibiting constitutive secretion (*ipaBc-ter*Δ*3, ipaDc-ter*Δ*3)*.

To compare the overall phenotype of all mutants, we plated them on CR plates with and without IPTG, which they need for *ipaD* expression (Fig. S6). Without IPTG, all strains were red due to absence of TCs and constitutive secretion, verifying they all made T3SSs. With IPTG, *ipaDwt ipaBwt* was orange, as expected for wild-type. All combinations of ipaB* and ipaD* mutants were white, indicating intact, CR-insensitive TCs and suggesting synergy between these mutations. In addition, all combinations of ipaB* or ipaD* mutants with *ipaDc-ter*Δ*3 or ipaBc-ter*Δ*3* mutants were orange to red, suggesting at best partial suppression of the constitutive secretion of *ipaDc-ter*Δ*3* or *ipaBc-ter*Δ*3*.

**Figure 5:**
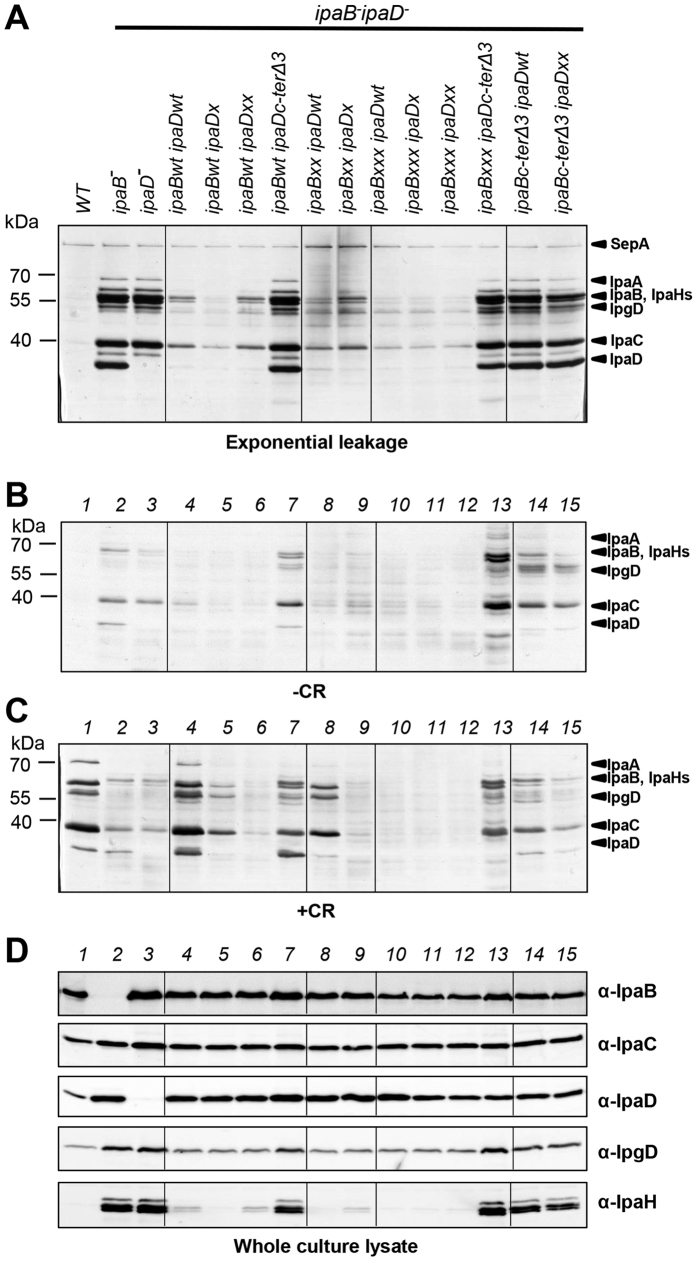
Characterization of *in trans* combined *ipaB* or *ipaD* mutants. *C*ombinations of ipaB* mutations and an IpaD C-terminal truncation, or vice versa, were studied in *ipaB*^−^ *ipaD*^−^, grown with 30 μM IPTG. (**A**) Exponential leakage. Protein secretion in response to absence (**B**) or presence (**C**) of CR. (**D**) Protein expression levels of translocators IpaB, IpaC and IpaD and late effectors IpaH and IpgD. Samples analyzed by Silver staining (**A–C**) or Western-blotted with the indicated antibodies (**D**). Results shown are representative of at least two independent experiments.

We next verified expression IpaB, IpaC and IpaD in these strains was similar to WT (Fig. 5D). We also assessed the levels of IpgD and IpaH. Indeed, the more the T3SS secretes, the higher the expression of IpaH and, to some extent, also of IpgD. The increased levels of IpgD and IpaH expression confirmed that, all combination of ipaD* or ipaB* mutants with *ipaBc-ter*Δ*3* or *ipaDc-ter*Δ*3*, respectively, were constitutive secretors.

No difference was observed in the TC composition of these mutants but one, *ipaBxxx ipaDc-ter*Δ*3* (Fig. 2C). Despite normal levels of IpaDc-terΔ3, it had lower levels of surface-localized IpaBxxx. Both *ipaBc-ter*Δ*3 ipaDwt* and *ipaBc-ter*Δ*3 ipaDxx*, show similar levels of IpaB but higher, if not significantly different, levels of IpaD.

Those “*” mutants that individually showed normal leakage, when combined with “*” or WT partners were confirmed to display the same phenotype and the same mutants in combination with constitutive secretors (*ipaBc-ter*Δ*3 & ipaDc-ter*Δ*3)* to display constitutive secretion. Thus, in *ipaBc-ter*Δ*3 ipaDxx,* contrary to what happened in *ipaBxxx_c-ter*Δ*3, ipaDxx* did not attenuate constitutive secretion of *ipaBc-ter*Δ*3,* although it formed compositionally normal tips (Fig. 5A, *lane 14 from left*). When *ipaBxxx* was combined with *ipaDc-ter*Δ*3* (Fig. 5A, *lane 13*) a constitutive secretor phenotype was also observed, due to lack of IpaBxxx and presence of *ipaDc-ter*Δ*3* in TCs. These data indicate a C-terminal deletion in IpaB bypasses the repressing effects of ipaD* mutations on secretion activation. This suggests IpaB’s C-terminus acts downstream of the upper part of the IpaD C-terminal helix. Furthermore, by comparison with the partial suppression of constitutive secretion seen with IpaBxxx_cterΔ3, lack of suppression of *ipaBc-ter*Δ*3* by *ipaDxx* suggests the globular domain of each protein can signal independently, via its own C-terminus.

Combination of mutants showing mild reductions in inducible secretion, *ipaBxx* and *ipaDx,* resulted in decreased CR-inducibility (Fig. 5B,C, *lanes 8 and 9*), whilst the combination of *ipaBxxx* with *ipaDx* resulted in abrogation of the latter’s inducibility (Fig. 5C, *lanes 10 and 11*). The combination of *ipaBxxx* and *ipaDxx in trans* did not enhance their already strong phenotypes (Fig. 5C, *lane 12*). Thus, mild IpaB* and IpaD* mutations are synergistic. These data, together with our TC reconstruction where IpaD and IpaB are juxtaposed^12^, support intermolecular communication between their globular regions during secretion activation. Finally, when the constitutive secretor *ipaBc-ter*Δ*3* was co-expressed with *ipaDxx*, another new phenotype was observed (Fig. 5B,C, *lane 15*): fast constitutive secretion as in *ipaBc-ter*Δ*3* (Fig. 5A) but as little CR-responsiveness as in *ipaDxx* (Fig. 5B,C). This suggests intermolecular crosstalk between the globular regions and the C-termini of these two molecules during CR sensing.

For *ipaBxxx ipaDc-ter*Δ*3,* in spite of the constitutive secretion described above, including of IpaD, we observed a reduction in the level of IpaD secreted without or without CR as compared to *ipaBwt ipaDc-ter*Δ*3* (Fig. 5B,C, *lane 13*). This was verified by western blotting (Fig. S7A). This indicates that IpaBxxx uniquely affects fast secretion of IpaDc-terΔ3.

### The activation signal travels from IpaB and IpaD to the needle

**Figure 6:**
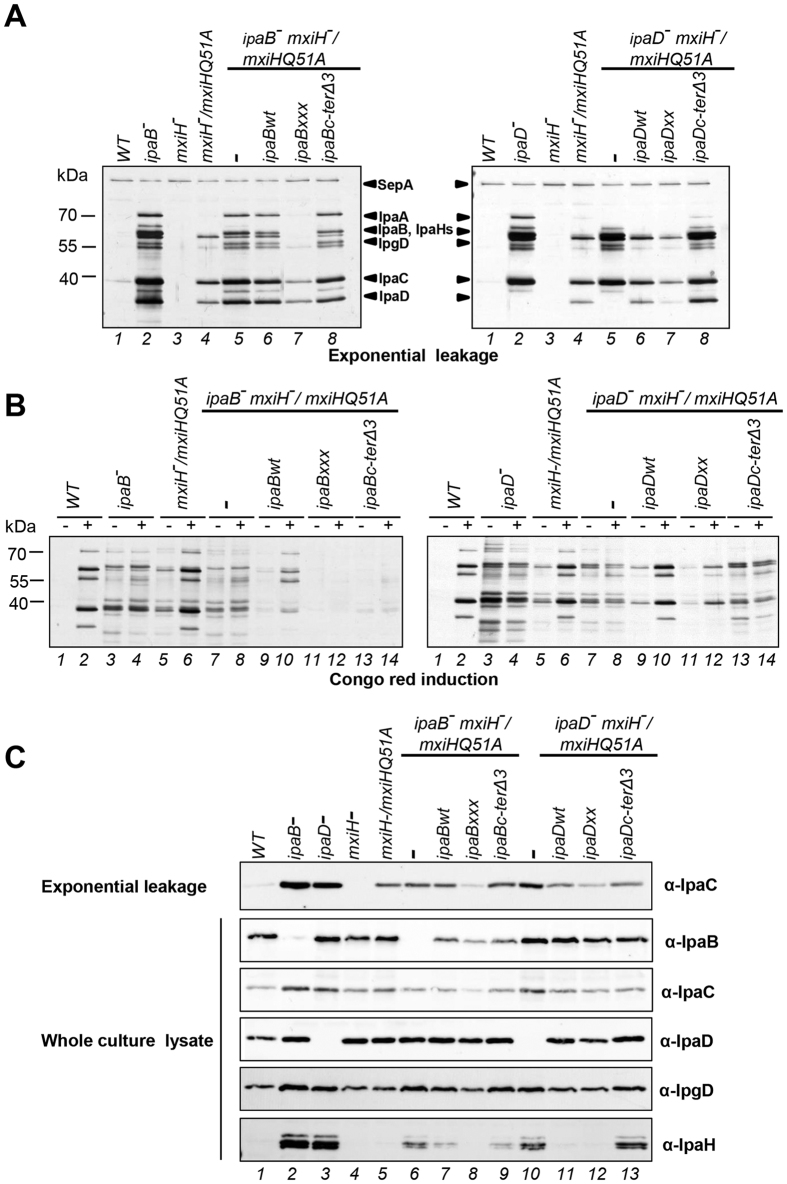
Analysis of combinations of *ipaB* or *ipaD* signaling mutants with *mxiHQ51A*. *ipaB* and *ipaD* mutant strains were grown with 20 μM IPTG and *mxiH*^−^/*mxiHQ51A* was grown in 25 μM IPTG. (**A**) Exponential leakage of indicated strains compared with WT, *ipaB*^−^*, ipaD*^−^ and *mxiH*^−^. (**B**) Protein secretion in response to CR of *ipaB* (*left*) and *ipaD* (*right*) mutants. (**C**) Expression levels of translocators IpaB, IpaC and IpaD and late effectors IpaH and IpgD. Samples were Silver stained (**A,B**) or blotted with the indicated antibodies (**C**). Results shown are representative of at least two independent experiments.

To examine how IpaB and IpaD interact with the needle component MxiH, we combined *ipaBxxx, ipaDxx, ipaBc-ter*Δ*3* and *ipaDc-ter*Δ*3* with slow constitutive secretor mutant *mxiHQ51A*^17^. This mutation is located in MxiH’s “head”, i.e. at the very top of the needle, where the C-termini of IpaD and IpaB probably interact MxiH^12^. Using FACS, we titrated the IPTG concentration necessary to obtain wild-type levels of IpaB, IpaD and MxiH in the *mxiHQ51A* background (Fig. S8). Then we confirmed the expression of IpaB, IpaC and IpaD in these strains was similar to WT (Fig. 6). We also assessed the expression of IpgD and IpaH.

*ipaB*^−^ and *ipaD*^−^ showed more IpaD and MxiH surface staining, respectively, by FACS (Fig. 2D). As neither *ipaB*^−^ nor *ipaD*^−^ show longer needles^6^, we investigated whether these mutants upregulate the number of T3SSs and hence TCs they express using electron microscopy analysis of negatively stained, osmotically shocked cells, as previously established^17^. We visualized 0.8 ± 1.3 (n = 14), 2.5 ± 2.3 (n = 19) and 3.7 ± 2.7 (n = 27) T3SS basal bodies on the periphery of WT, *ipaB*^−^ and *ipaD*^−^ cells, respectively. Therefore, as demonstrated by a Student’s test after ANOVA, *ipaB*^−^ and *ipaD*^−^ possess similar number of basal bodies but both possess significantly more than wild-type (p = 0.0162 and 0.0004, respectively). This suggests that the fast constitutive secretion state leads to a 3–4 fold increase the number of T3SS basal bodies. In addition, *mxiHQ51A* showed a reduction in IpaB surface staining relative to wild-type. This was not previously observed^12^,^13^, but might contribute to increased leakage in this mutant. In a *mxiHQ51A* background, *ipaBc-ter*Δ*3* and *ipaDc-ter*Δ*3* showed a significant absence of IpaB, but not of IpaD (Fig. 2D). However, in a *mxiHwt* background these two mutants show normal TC composition (Fig. 2B). This indicates short C-terminal deletions in IpaDc-terΔ3 or IpaBc-terΔ3 adversely affect IpaB’s interaction with MxiH with a point mutation in its head. Finally, in a *mxiHQ51A* background, *ipaBxxx* showed slightly higher (significant at p = 0.05 but not at p = 0.02) and *ipaDxx* showed higher levels of IpaB surface staining, respectively, suggesting these mutations stabilize IpaB at the TC. These data indicate the IpaB C-terminus interacts with MxiHQ51 and this is affected by mutations in the alacoil and in the C-terminus of IpaD.

No expression of IpaH was detected in *mxiHQ51A* whole cell extract although this mutant is a slow constitutive secretor (Fig. 6C). This suggests the level of constitutive secretion in *mxiHQ51A* is not strong enough to activate *ipaH* transcription. Furthermore, *ipaB*^−^, *ipaBc-ter*Δ*3, ipaD*^−^ *and ipaDc-ter*Δ*3* when each combined *in trans* with *mxiHQ51A* show similarly increased levels of IpgD and IpaH, confirming their fast constitutive secretion.

As expected from their tip composition, *ipaBc-ter*Δ*3* and *ipaDc-ter*Δ*3* displayed constitutive secretor phenotypes when combined with *mxiHQ51A* (Fig. 6A *left and right* panels). However, for *ipaDc-ter*Δ*3* this level was higher than in *ipaDwt.* In *ipaDc-ter*Δ*3 mxiHQ51A,* IpaB is lost from TCs whereas both proteins are still present intracellularly, again suggesting the former is the cause of fast constitutive secretion. The differing levels of leakage (Fig. 6A,C, *top row*) displayed by *mxiH*^−^/*mxiHQ51A* versus *mxiH*^−^ *ipaB*^−^/*mxiHQ51A ipaBwt* indicate that in the latter, complementation by *ipaB* is only partial (Fig. 6A, *left, lanes 4–6*), perhaps because its expression level is lower than in the former (Fig. 6C, *lanes 5–7*). However, *ipaBxxx* suppresses the leakage seen in *mxiH*^−^ *ipaB*^−^/*mxiHQ51A ipaBwt* (Fig. 6A, *left, lanes 7*). Full complementation occurred in *mxiH*^−^ *ipaD*^−^/*mxiHQ51A ipaDwt* (Fig. 6A, *right, lanes 4–6*) and suppression of leakage by *ipaDxx* was also seen. That *ipaBxxx* and *ipaDxx* both stabilize IpaB in the TC in an *mxiHQ51A* background (Fig. 2D) may explain the leakage reduction. Both *ipaBxxx* and *ipaDxx* suppress *mxiHQ51A* more strongly under inducible conditions (Fig. 6B, *both sides, lanes 9–12*). This indicates that IpaD and IpaB signal to MxiH, initially via their globular domains.

Under non-induced conditions *ipaBc-ter*Δ*3 mxiHQ51A* released IpaC and IpaD in normal amounts, but not IpaB (Fig. S7B), although IpaB is secreted in *ipaBc-ter*Δ*3*^23^. In addition *ipaDxx mxiHQ51A* could leak IpaD but not secrete it inducibly (compare Fig. 6A, *right, lane 7* to 6B, *right, lanes 10 & 12*). Together with the data showing that *ipaBxxx* affects secretion of IpaDc-terΔ3, this suggests IpaB and IpaD can sense each other’s status in the TC and regulate aspects of their secretion.

**Figure 7:**
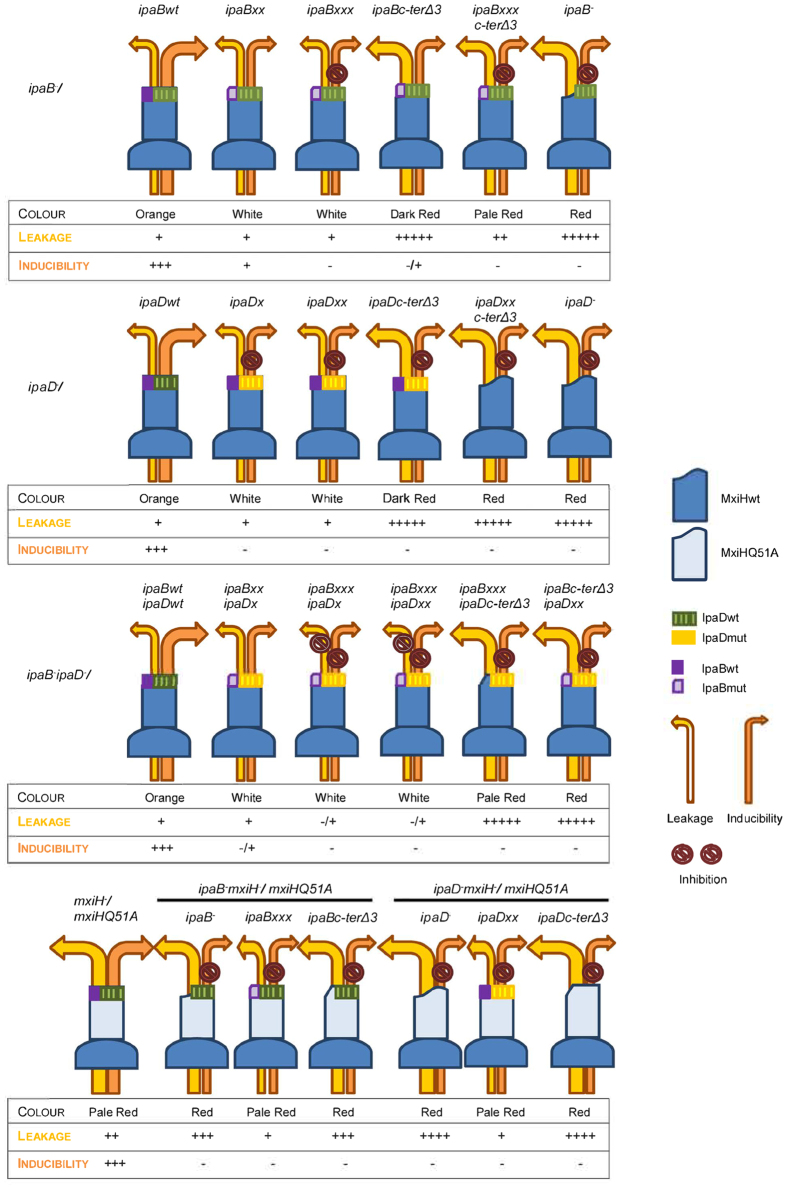
Schematic summary table of phenotypes of key mutants studied. Illustration of the effect of *ipaB* and *ipaD* mutations on the phenotype of the studied and/or newly constructed strains. The term “Color” is based in the colony color displayed when grown on TCS agar supplemented with 100 μg/ml CR (as in Fig. S6). Leakage and inducibility have been represented according to the phenotypes shown in Silver stained gels of exponential leakage and protein secretion under CR induction (Figs 1 and 3,4,5,6). Finally, tip composition has been drawn in accordance with FACS results (Fig. 2).

All *ipaB* and *ipaD* mutations studied are epistatic over *mxiHQ51A* in that they change TC composition and/or affect aspects of secretion regulation. Given their location atop needles^12^, this demonstrates both proteins regulate secretion upstream of the needle, i.e. from the TC. Our overall results, summarized in Fig. 7, now better mechanistic understanding of the TC’s intriguing functionalities.

## Discussion

### IpaB is involved in sensing the secretion activation signal at the needle tip

All ipaB* mutants are partially defective in CR-sensing although they show normal TC composition. Furthermore, they are stably expressed, able to prevent premature secretion and do not have intrinsic secretion defects. IpaB∆2-20’s cytoplasmic sequestration provokes maximal constitutive secretion and uninducibility. There is presently no evidence that IpaB exerts any direct regulatory function inside the bacterium, although its secretion is signaled by its “empty” chaperone. This is supported by the phenotype of *ipaBc-ter*Δ*3*, which has a normal N-terminus but is still a constitutive secreter because of its needle interaction defect. Short C-terminal truncations in IpaB may alter its conformation at needle tips, leading to constitutive secretion with some inducibility^21^. Thus, IpaB’s presence at the tip is essential for T3SS regulation and inducibility. Therefore, 1) our IpaB* mutants are acting from the TC and 2) TCs containing only pentameric IpaD are a secretion-deregulated assembly intermediate that exists prior to IpaB recruitment or due to the temporary loss of the hydrophobic IpaB from the needle tip^12^.

Some combinations of ipaB* mutants display additive phenotypes. The triple mutant *ipaBxxx* abrogates inducibility although it is stably at the tip. The fast constitutive secretor and partially inducible phenotype of *ipaBc-ter*Δ*3* was reduced by *in cis* addition of *ipaBxxx.* These two sets of mutations are in different regions but, when localized in the same molecule, they alter each other’s effect. Observations of synergism and epistasis support the notion that the activation signal travels within IpaB.

### IpaB’s involvement in events beyond host cell sensing

All previously published *ipaB* mutants carry substantial deletions, leading to pleiotrophic effects^23^,^24^,^41^. Here, we used an unbiased method to obtain single mutations, focusing on IpaB’s role in secretion regulation. Selected mutants were also used to analyze IpaB’s interaction with eukaryotic cells. *ipaBN264I* is strongly impaired in hemolysis and invasion because it is unable to insert IpaB and IpaC in RBC membranes. *ipaBQ108L* also displays substantial reductions in membrane-inserted IpaB and IpaC. In contrast, *ipaBN116Y* shows no reduction in hemolysis or invasion although it displays less membrane-inserted IpaB and IpaC. Why the latter two mutants are affected in these steps is not clear. *ipaBN85I* is mildly affected in CR-sensitivity, hemolysis and translocon insertion but to a greater extent in invasion. This mutant might thus be defective in events after translocon insertion, such as effector release^17^,^53^.

### Role of IpaB’s alacoil in signal transduction

All but one ipaB* mutation are located in its alacoil^22^. In this region, IpaB and its *Salmonella* SPI1 T3SS homolog SipB show structural similarity with the receptor-binding domain of E2, E3/E9 and Ia colicins^22^. Colicins are secreted by some strains of *Escherichia coli* and lethal for others^54^. The colicin sequence similarity does not extend into the amphipathic helix and two transmembrane regions of the IpaB family, although the topologically equivalent portion of pore-forming colicins (such as Ia) has similar features. These hydrophobic features are shared too with the Bcl-2 family of apoptosis regulators^55^ and with the membrane insertion domains of diphtheria toxin^56^, which both also form membrane pores. Recently, the structure of central region of an IpaB homolog from *Aeromonas*, was solved in combination with its chaperone. AopB’s fold is very reminiscent of the hydrophobic region of pore-forming colicins and Bcl-2 proteins, with the hydrophilic and amphipathic helices wrapping around and shielding the hydrophobic ones^57^. This suggests IpaB/SipB adapted mechanisms used by pore-forming toxins to insert into membranes.

The colicin E9 alacoil region opens up for it to insert into target membranes^58^. Perhaps IpaB also “opens up” upon insertion of its hydrophobic regions into host membranes, thus initiating the signaling cascade (Fig. S9)? The clustered location of our “*” mutants within the alacoil and their synergistic effects suggest conformational changes in this region trigger secretion activation. However, if preventing such an alteration were how our new CR-insensitive mutations act, the IpaB* mutants should to be unable to sense host cells as well. More likely therefore, and in view of their synergism with our IpaD* mutants, the mutations render the IpaB-IpaD interface more resistant to disruption by CR, whilst the host cell is sensed differently. Given IpaB’s physically distal position in the TC, this may occur through IpaB alone, initially via an area other than its alacoil. However, the signal would be transduced through the alacoil and via IpaD since *ipaBxx* and ipaD* mutants are largely insensitive to host cells. *ipaBN264I*, in the amphipathic helix, impairs insertion of IpaB in host membranes and is also partially CR-insensitive. It therefore highlights a connection between changes in the amphipathic helix, which lies parallel to the membrane in liposome-inserted IpaB^52^,^59^, and signal transmission via the alacoil, where the other CR-insensitive mutations map.

### Directionalities for signal transduction

Single amino acid substitutions in IpaB deregulate secretion activation. Combining deregulated mutants demonstrated intramolecular communication within IpaB (Figs 3D,F and 4) and intermolecular communication between TC components (Fig. 5). Moreover, *ipaBxxx* and *ipaDxx* both suppress *mxiHQ51A* constitutive secretion (Fig. 6). This indicates IpaB and IpaD act in the same pathway and via MxiH to regulate secretion activation. Considering TC morphology^12^, we conclude IpaB is the major host cell sensor while IpaD works it with to transmit the activation signal down the needle.

How is the signal transmitted down the TC to the needle? Localization of our IpaB mutations to the amphipathic helix and alacoil suggests these regions act coordinately. We uncovered further crosstalk between different regions of IpaB by combining ipaB* alacoil mutations *in cis* with some in the IpaB C-terminus^23^. IpaB-IpaD, IpaB-MxiH, IpaD-MxiH (this study and^28^,^29^) and MxiH-MxiH^17^ interactions are all important. IpaD-IpaD interactions are difficult to probe. Without an atomic resolution model for the TC and needle, the mechanism of this signal transduction event is unapproachable in further detail.

## Methods

### Bacterial strains and culture

Bacterial strains and plasmids used are listed in Tables 1 and 2. *Shigella flexneri* strains were maintained on Congo red (CR, 100 μg/ml; Serva) agar plates and grown at 37 °C in Trypticase Soy Broth (Becton Dickinson) supplemented with antibiotics when necessary (100 μg/ml ampicillin, 50 μg/ml kanamycin, 10–20 μg/ml chloramphenicol, 5 μg/ml tetracycline). IPTG and arabinose were used at the concentrations indicated in the figure legends.

### Construction of *ipaBΔ2-20*

PCR site-directed mutagenesis using pDR1^23^ as a template was carried out with Pfx Platinum polymerase (Invitrogen). *ipaB* was amplified using forward primers 1 and 2 (Table 3) to create *ipaBwt* and *ipaB*Δ*2-20*, respectively. Primer 5 was used as reverse primer. After digestion with HindIII and PstI, fragments were cloned into pUC19, thereby removing eight additional N-terminal amino acids introduced by earlier subcloning^60^ that could have an undesirable effect on the signal peptide and giving rise to plasmids pIMC34 and pIMC35. This vector had the same levels of IpaB expression as pDR1, which also yields functional IpaB protein^60^ and which was previously used to isolate the point mutants below.

To assess the level of IpaB necessary for complementation a new plasmid was constructed where ipaB was placed under the control of an arabinose inducible promoter, giving rise to pIMC30. We used pBAD_myc_His cut by NcoI and PstI to clone *ipaB* amplified by PCR with primers 4 and 18.

### Construction of the *ipaB* mutant libraries

PCR mutagenesis was carried out with Taq DNA polymerase (New England Biolabs) using error prone reaction conditions (3 mM and 4 mM Mg^2+^), primers 3 and 4 and pDR1 as template. PCR fragments were purified, digested with HindIII and PstI and ligated into plasmid pDR1^23^. Ligation mixtures were electroporated into *E. coli* DH5α carrying an empty pACT3 to produce LacI and repress otherwise toxic *ipaB* expression or into XL2 for the same reason. Transformations were incubated for 16h at 37 °C, plasmid DNA was then extracted and kept at −20 °C.

### Screening of *ipaB* mutant libraries

To identify non-inducible mutants, DNA from each mutant library was electroporated into *ipaB*^−^ (red colony) and screened for white colonies on TCS agar plates containing 100 μg/ml CR. Putative ‘white’ colonies were selected, their plasmids isolated and retransformed into the *ipaB*^−^ mutant to ensure the white color was not due to loss-of-function mutations elsewhere within the T3SS-encoding operons. Candidate plasmids were sequenced to identify the mutation(s) responsible for the mutant phenotype (Table 2).

### Combination of double and triple *ipaB* mutants

We constructed the new mutants using two-step PCR followed by fragment purification, digestion with HindIII and SpeI and subsequent cloning into digested pDR1. As mutations in pIMC40 (*ipaBN85I, K93N*) *and* pIMC41 (*ipaBQ108L, N116Y*) are close they were introduced together in the PCR primers. The first step of PCR consisted in two reactions, one reaction used primers 3 and 6 and second reaction primers 5 and 7 for pIMC40, where primers 6 and 7 overlap and contain the mutation, and pairs 3 and 9 and 5 and 8 for pIMC41, where primers 8 and 9 overlap and contain the mutation; pDR1 was used as template. The two PCR fragments were used as a template for the second step PCR, which was performed using primers 3 and 5 for both pIMC40 and pIMC41.

For pIMC42 (*ipaBK150E, K188E*), as both mutations are farther apart, mutation K188E was introduced by two-step PCR into pIMC4 already encoding K150E. The first step PCR was carried out with primer pair 3 and 11 and primer pair 5 and 10. The second step was as for pIMC40 and pIMC41, with primers 3 and 5. The triple mutant pIMC43 (*ipaBQ108L, N116Y, K150E*) was constructed as for pIMC41 but using pIMC4 as template.

*ipaBxx, ipaBxxx*, and *ipaBwt* were later introduced into pUC18_oc plasmid^12^, which is insensitive to LacI, to combine them *in trans* with the IPTG inducible plasmid pACT3. The *ipaB* mutants were amplified by PCR using primers 12 and 13, using pDR1, pIMC42 and pIMC43 as templates. PCR fragments were then digested with NdeI and BamHI and cloned into pUC18_oc given rise to plasmids pIMC46, pIMC51 and pIMC57.

### Construction of truncated *ipaBΔ3* and *ipaDΔ3* and derivatives

We cloned *ipaB*Δ*3* into pUC18-oc by digesting pDR2 with SpeI and PstI and ligated into pIMC51 given rise to pIMC56. The SpeI-PstI digested fragment was also cloned into pIMC43 generating the new plasmid pIMC47 containing *ipaBxxx in cis* with *ipaB*Δ*3.*

We also constructed a series of *ipaD* mutants in pUC18-oc^12^. We cloned the new *ipaD* constructs into pDR6-oc plasmid. We first reintroduced the native C322 into *ipaD* by digesting pipaD with EcoRV/PstI and ligating into pDR6-oc, creating pIMC61. We then constructed pIMC62 by ligating *ipaDN186Y, K291I* digested from pIMA237 with EcoRV and PstI into pDR6-oc. Then we made pIMC63 by deleting the last 3 aa of IpaD by PCR using pipaD as template and primers 14 and 15. PCR fragment was digested with EcoRV and BamHI and ligated into pDR6-oc. These primers were also used to obtained *ipaDxxD3* by PCR, although in this case, pIMA237 was used as a template. The PCR fragment was digested and ligated into pDR6-oc given rise to pIMC64.

### Combination of *ipaB* and *ipaD* mutants

To combine *ipaB* and *ipaD in trans* we constructed a series of *ipaD* mutants in pACT3. By PCR, using primers 16 and 17, we amplified *ipaDx* and *ipaDxx* from pIMA233 and pIMA237, respectively. PCR fragments were digested and ligated into pACT3 giving rise to pIMC58 and pIMC60. To amplify *ipaD*Δ*3* from pIMAC49, we used primers 15 and 16. After digestion with SacI/BamHI and ligation into pACT3, pIMC59 was obtained. *ipaDwt* was amplified from pipaD by PCR using primers 16 and 17. The PCR fragment was digested and cloned into pACT3 giving rise to pIMA246.

All plasmids created were verified by sequencing (Eurofins).

### Expression of *mxiQ51A in trans* with *ipaB* and *ipaD* mutants

A series of *ipaB* and *ipaD* mutants cloned into pUC18-oc were co-expressed *in trans* with *mxiHQ51A,* expressed from pACT3.

### Analysis of protein synthesis and secretion

#### Protein expression levels

Strains were grown at 37 °C until mid-exponential phase (OD_600_ = 1) was reached. Samples of the cultures were denatured in Laemmli sample buffer. Samples from equivalent cell numbers were separated by SDS-PAGE and Western blotted.

#### Exponential leakage

Strains were grown until OD_600_ = 1. Cultures were centrifuged at 15,000 g for 10 min at 4 °C and supernatants from equivalent cell numbers were subjected to SDS-PAGE and Silver-stained (Silver Xpress kit, Invitrogen) or Western blotted.

#### Congo red induction

Bacteria collected during mid-exponential growth were resuspended at OD_600_ = 5 in phosphate-buffered saline (PBS). CR (Serva) was added at 100 μg/ml. After incubation at 37 °C for 15 min, the samples were centrifuged at 15,000 g for 10 min at 4 °C and the supernatants separated by SDS-PAGE and Silver-stained or Western blotted.

#### Western blots

Proteins separated by SDS-PAGE were transferred onto PVDF membrane (Immobilon FL, Millipore) and hybridized with anti-IpaB, IpaC, IpaD, IpgD and IpaH^43^. Fluorescent secondary antibodies (goat anti-rabbit-Alexa680, Invitrogen; goat anti-rabbit-DyLight800 and goat anti-mouse-DyLight800, Pierce) were visualized and quantified using an Odyssey infrared imaging system (Li-Cor).

### Characterization of the cellular interactions of *ipaB* mutants

#### Contact-mediated hemolysis

Contact hemolysis of sheep red blood cells (RBC) was performed as described previously^23^. PBS (Sigma) was used throughout.

#### Red blood cell membrane isolation

RBC membrane isolation was performed as described previously^24^.

#### Invasion assays

Gentamycin protection assays, were performed as previously described^24^ with small modifications. During the experiment HeLa cells were incubated in DMEM high glucose (Sigma) complemented with heat inactivated 10% fetal bovine serum (Sigma) to minimize cell stress caused by FBS depletion.

#### FACS analysis

FACS was used to assess the presence of IpaB, IpaD and MxiH on the *Shigella* surface as previously described^12^.

#### Confocal microscopy

HeLa cells were grown to 70% confluence on poly-lysine coverslips. Cells were infected with log phase *Shigella* at a MOI of 20. An Afa1-expressing bacteria strain was used to increase bacterial adhesion^61^. The samples were centrifuged for 10 min at 900g at room temperature to synchronise adhesion, then incubated for 6 min at 37 °C to initiate interaction with host cells. Cells were fixed with 2% PFA, blocked with 3% w/v BSA in PBS and then immunostained for 1 h at room temperature with anti-IpaD, anti-IpaH and anti-MxiH (see FACS section for santibody details). Host cell actin was stained with Alexa Fluor 488 Phalloidin (Life Technology) according to the manufacturer’s instructions. Samples were mounted with Mowiol. Images were taken using a Leica SP5-II confocal laser scanning microscope using a 63X oil objective.

#### Analysis of T3SS basal body abundance by electron microscopy

Bacterial cells were processed as described in Kenjale *et al*.^17^ to obtain cell ghosts. Electron micrographs were recorded on a FEI Tecnai T12 transmission electron microscope (TEM), operating at 100 kV. Images were acquired at a nominal magnification of 26,000X.

## Additional Information

**How to cite this article**: Murillo, I. *et al*. Genetic Dissection of the Signaling Cascade that Controls Activation of the *Shigella* Type III Secretion System from the Needle Tip. *Sci. Rep.* **6**, 27649; doi: 10.1038/srep27649 (2016).

## Supplementary Material

Supplementary Information

## Figures and Tables

**Table 1:** Bacterial strains used in this study.

**Strain name**	**Genotype**	**Reference**
Wild-type	Wild-type M90T, serotype 5a	62
SC301	M90T/pIL22, a plasmid encoding the *E. coli* afimbrial adhesin AFA I	61
SF620 or *ipaB*^−^	Δ*ipaB::aphA-3 mutant*	63
SF622 or *ipaD*^−^	Δ*ipaD::aphA-3 mutant*	63
SH116 or *mxiH*^−^	Δ*mxiH::aphA-3 mutant*	7
*ipaB*^−^ *ipaD*^−^	Double mutant Δ*ipaB::aphA-3* Δ*ipaD::tetRA*	21
*ipaB*^−^*/ipaB or ipaB*^−*/*+^	*ipaB*^−^/pDR1	23
*ipaB*^−^*/ipaBwt*	*ipaB*^−^*/*pIMC34	This study
*ipaB*^−^*/ipaB*Δ*2-20*	*ipaB*^−^/pIMC35	This study
*ipaBN85I*	*ipaB*^−^/pIMC24 (isolated from *ipaB* library)	This study
*ipaBK93N*	*ipaB*^−^/pIMC5 (isolated from *ipaB* library)	This study
*ipaBQ108L*	*ipaB*^−^/pIMA254 (isolated from *ipaB* library)	This study
*ipaBN116Y*	*ipaB*^−^/pIMC18 (isolated from *ipaB* library)	This study
*ipaBK150E*	*ipaB*^−^/pIMC4 (isolated from *ipaB* library)	This study
*ipaBK188E*	*ipaB*^−^/pIMC1 (isolated from *ipaB* library)	This study
*ipaBN264I*	*ipaB*^−^/pIMA255 (isolated from *ipaB* library)	This study
*ipaBN85I, K93N*	*ipaB*^−^/pIMC40	This study
*ipaBQ108L, N116Y*	*ipaB*^−^/pIMC41	This study
*ipaBK150E, K188E* aka *ipaBxx*	*ipaB*^−^/pIMC42	This study
*ipaBQ108L, N116Y, K150E aka ipaBxxx*	*ipaB*^−^/pIMC43	This study
*ipaB*Δ*578-580* aka *ipaBc-ter*Δ*3*	*ipaB*^−^/pDR2	23
*ipaBQ108L, N116Y, K150E,* Δ*578-580* aka *ipaBxxxc-ter*Δ*3*	*ipaB*^−^/pIMC47	This study
*ipaD-/ipaD or ipaD*^−*/*+^	*ipaD*^−^*/pipaD*	44
*ipaD-/ipaDK291I*	*ipaD*^−^*/*pIMA233	21
*ipaD-/ipaDQ299I*	*ipaD*^−^*/*pIMA236	21
*ipaB*^−^ *ipaD*^−^*/ipaBN85I*	*ipaB*^−^ *ipaD*^−^*/*pIMC24	This study
*ipaB*^−^ *ipaD*^−^*/ipaBK93N*	*ipaB*^−^ *ipaD*^−^*/*pIMC5	This study
*ipaB*^−^ *ipaD*^−^*/ipaBQ108L*	*ipaB*^−^ *ipaD*^−^*/*pIMA254	This study
*ipaB*^−^ *ipaD*^−^*/ipaBN116Y*	*ipaB*^−^ *ipaD*^−^*/*pIMC18	This study
*ipaB*^−^ *ipaD*^−^*/ipaBK150E*	*ipaB*^−^ *ipaD*^−^*/*pIMC4	This study
*ipaB*^−^ *ipaD*^−^*/ipaBK188E*	*ipaB*^−^ *ipaD*^−^*/*pIMC1	This study
*ipaB*^−^ *ipaD*^−^*/ipaBN264I*	*ipaB*^−^ *ipaD*^−^*/*pIMA255	This study
*ipaD*^−^*/ipaDwt*	*ipaD*^−^*/pIMC61*	This study
*ipaD*^−^*/ipaDN186Y, K291I* aka *ipaDxx*	*ipaD*^−^*/*pIMC62	This study
*ipaD*^−^*/ ipaD*Δ*330-332* aka *ipaDc-ter*Δ*3*	*ipaD*^−^*/*pIMC63	This study
*ipaD*^−^*/ ipaDN186Y, K291I,* Δ*330-332* aka *ipaDxxc-ter*Δ*3*	*ipaD*^−^*/*pIMC64	This study
*ipaB*^−^ *ipaD*^−^*/ipaBwt*	*ipaB*^−^ *ipaD*^−^*/*pIMC51	This study
*ipaB*^−^ *ipaD*^−^*/ipaDwt*	*ipaB*^−^ *ipaD*^−^*/*pIMA246	This study
*ipaB*^−^ *ipaD*^−^*/ipaBwt ipaDwt*	*ipaB*^−^ *ipaD*^−^*/*pIMC51 pIMA246	This study
*ipaB*^−^ *ipaD*^−^*/ipaBwt ipaDx*	*ipaB*^−^ *ipaD*^−^*/*pIMC51 pIMC60	This study
*ipaB*^−^ *ipaD*^−^*/ipaBwt ipaDxx*	*ipaB*^−^ *ipaD*^−^*/*pIMC51 pIMC58	This study
*ipaB*^−^ *ipaD*^−^*/ipaBwt ipaDc-ter*Δ*3*	*ipaB*^−^ *ipaD*^−^*/*pIMC51 pIMC59	This study
*ipaB*^−^ *ipaD*^−^*/ipaBxx ipaDwt*	*ipaB*^−^ *ipaD*^−^*/*pIMC57 pIMA246	This study
*ipaB*^−^ *ipaD*^−^*/ipaBxx ipaDx*	*ipaB*^−^ *ipaD*^−^*/*pIMC57 pIMC60	This study
*ipaB*^−^ *ipaD*^−^*/ipaBxxx ipaDwt*	*ipaB*^−^ *ipaD*^−^*/*pIMC46 pIMA246	This study
*ipaB*^−^ *ipaD*^−^*/ipaBxxx ipaDx*	*ipaB*^−^ *ipaD*^−^*/*pIMC46 pIMC60	This study
*ipaB*^−^ *ipaD*^−^*/ipaBxxx ipaDxx*	*ipaB*^−^ *ipaD*^−^*/*pIMC46 pIMC58	This study
*ipaB*^−^ *ipaD*^−^*/ipaBxxx ipaDc-ter*Δ*3*	*ipaB*^−^ *ipaD*^−^*/*pIMC46 pIMC59	This study
*ipaB*^−^ *ipaD*^−^*/ipaBc-ter*Δ*3 ipaDwt*	*ipaB*^−^ *ipaD*^−^*/*pIMC56 pIMA246	This study
*ipaB*^−^ *ipaD*^−^*/ipaBc-ter*Δ*3 ipaDxx*	*ipaB*^−^ *ipaD*^−^*/*pIMC56 pIMC58	This study
*mxiH*^−^*/mxiHQ51A*	*mxiH*^−^*/ pmxiHQ51A*	15
*ipaB*^−^ *mxiH*^−^	*Double mutant* Δ*ipaB:: tetRA* Δ*mxiH:: aphA-3*	43
*ipaB*^−^ *mxiH*^−^*/mxiHQ51A*	*ipaB*^−^ *mxiH*^−^*/* pmxiHQ51A	This study
*ipaB*^−^ *mxiH*^−^*/ ipaBwt mxiHQ51A*	*ipaB*^−^ *mxiH*^−^*/*pIMC51 pmxiHQ51A	This study
*ipaB*^−^ *mxiH*^−^*/ipaBxxx mxiHQ51A*	*ipaB*^−^ *mxiH*^−^*/*pIMC46 pmxiHQ51A	This study
*ipaB*^−^ *mxiH*^−^*/ ipaBc-ter*Δ*3 mxiHQ51A*	*ipaB*^−^ *mxiH*^−^*/*pIMC56 pmxiHQ51A	This study
*ipaD*^−^ *mxiH*^−^	*Double mutant* Δ*ipaD:: tetRA* Δ*mxiH:: aphA-3*	12^a^
*ipaD*^−^ *mxiH*^−^*/mxiHQ51A*	*ipaD*^−^ *mxiH*^−^*/*pmxiHQ51A	This study
*ipaD*^−^ *mxiH*^−^*/ipaDwt mxiHQ51A*	*ipaD*^−^ *mxiH*^−^*/*pIMA246 pmxiHQ51A	This study
*ipaD*^−^ *mxiH*^−^*/ipaDxx mxiHQ51A*	*ipaD*^−^ *mxiH*^−^*/*pIMC58 pmxiHQ51A	This study
*ipaD*^−^ *mxiH*^−^*/ipaDc-ter*Δ*3 mxiHQ51A*	*ipaD*^−^ *mxiH*^−^*/*pIMC59 pmxiHQ51A	This study

^a^We have noticed that all strains made with this background express less, and hence secrete little IpaA. This may be due to the manner in which *ipaD*, which lies directly upstream of *ipaA*, was inactivated in them. However, this has no bearing on the study described here.

**Table 2:** Plasmids used in this study.

**Plasmid**	**Description**	**Reference**
pDR1	pUC19::*ipaB*	23
pDR2	pDR1::*ipaB*Δ*578-580* aka *ipaB*Δ*3*	23
pIMA254	pDR1::ipaBQ108L	This study
pIMA255	pDR1::ipaBN264I	This study
pIMC34	pUC19*::ipaB* (no8aa extras and RBSbad)	This study
pIMC35	pIMC34*::ipaB*Δ*2-20*	This study
pIMC1	pDR1::*ipaBK188E*	This study
pIMC4	pDR1::*ipaBK150E*	This study
pIMC5	pDR1::*ipaBK93N*	This study
pIMC18	pDR1::*ipaBN116Y*	This study
pIMC24	pDR1::*ipaBN85I*	This study
pIMC40	pDR1::*ipaBN85I, K93N*	This study
pIMC41	pDR1::*ipaBQ108L, N116Y*	This study
pIMC42	pDR1::*ipaBK150E, K188E*	This study
pIMC43	pDR1::*ipaBQ108L, N116Y, K150E*	This study
pUC18_oc	Modified pUC18 insensitive to LacI	12
pDR6_oc	pUC18_oc::ipaDC322S	12
pIMC46	pUC18_oc::*ipaBQ108L, N116Y, K150E*	This study
pIMC47	pDR1::*ipaBQ108L, N116Y, K150E,* Δ*578-580*	This study
pIMC49	pUC18::*ipaD*Δ*330-332*	This study
pIMC51	pUC18_oc::*ipaBwt*	This study
pIMC56	pUC18_oc::*ipaB* Δ*578-580*	This study
pIMC57	pUC18_oc::*ipaBK150E, K188E*	This study
pipaD	pUC18::*ipaD*	44
pIMA233	pUC18::*ipaDK291I*	21
pIMA236	pUC18::*ipaDQ299L*	21
pIMA237	pUC18::*ipaDN186Y, K291I*	21
pIMA246	pIMA246::*ipaDwt*	This study
pIMC58	pIMA246::*ipaDN186Y, K291I*	This study
pIMC59	pIMA246::*ipaD*Δ*330-332*	This study
pIMC60	pIMA246::*ipaDK291I*	This study
pIMC61	pUC18_oc::*ipaD*	This study
pIMC62	pUC18_oc::*ipaDN186Y, K291I*	This study
pIMC63	pUC18_oc::*ipaD*Δ*330-332*	This study
pIMC64	pUC18_oc::*ipaDN186Y, K291I,* Δ*330-332*	This study
pmxiHQ51A	pACT3::*mxiHQ51A*	15
pIMC30	pBAD_myc_HisA::*ipaB*	This study

**Table 3:** Primers used in this study.

**#**	**Primer name**	**Sequence**^**a**^
1	ipaBwt_no88a_RBSbad Fwd	GCGAAGCTTCAGGAGGAATTAACCATGCATAATGTAAGCACCACAAC
2	ipaBΔ2-20_no88a_RBSbad Fwd	GCGAAGCTTCAGGAGGAATTAACCATGGAGCTTGGAGACAATACTATC
3	ipaB HindIII Fwd	GGGGAAGCTTGATGCATAATGTAAGCACC
4	ipaB PstI Rev	GGGGCTGCAGTCCTTATTTGTATCAAGCAG
5	ipaB SpeI Rev	GCGCACTAGTTGAATAAAGGTTGCC
6	ipaB N85I-K93N Rev	TTAATGCAGTTAAAGAGTTTTCACCGAGTATTTGAATAAGGATTCCAATTAAAAGC
7	ipaB N85I & K93N Fwd	GCTTTTAATTGGAATCCTTATTCAAATACTCGGTGAAAACTCTTTAACTGCATTAAC
8	ipaBQ108L & N116I Fwd	CTTGGAAGTCCCTGCAACAGGCAAGACAGCAAAAATACCTAGAATTCTC
9	ipaBQ108L & N116I Rev	GAGAATTCTAGGTATTTTTGCTGTCTTGCCTGTTGCAGGGACTTCCAAG
10	ipaB K188E Fwd	CTCACTATCGAAAAAGACGCAGCAGTTAAAGAC
11	ipaB K188E Rev	GTCTTTAACTGCTGCGTCTTTTTCGATAGTGAG
12	ipaB_NdeI Fwd	GCGCATATGCATAATGTAAGCACCACAACCACTG (pUC18-oc)
13	ipaB_BHI Rev	CGCGGATCCTCAAGCAGTAGTTTGTTGC (pUC18-oc)
14	ipaD_EcoRV_Fwd	GGAGATCAAATGATATCTCATAG (pUC18-oc)
15	ipaD_Δ330-332-BHI_Rev	CGCGGATCCTCAAAAAAGTTTATCTGTATCTGTAC (pUC18-oc)
16	ipaD_SacI_Fwd	CGAAAAGAGCTCTAAGGAAATAACCATGAATATAACAACTCTGACTAATAG (pACT3)
17	ipaD_BHI_Rev	CGCGGATCCTCAGAAATGGAGAAAAAG (pACT3)
18	ipaB_NcoI Fwd	CGCGCCATGGATAATGTAAGCACCACAACCACTGG

^a^Restriction sites are underlined.
