# Supplementary material for: Genetic Dissection of the Signaling Cascade that Controls Activation of the Shigella Type III Secretion System from the Needle Tip
Source: Sci Rep. 2016 Jun 9;6:27649. doi: 10.1038/srep27649 (PMC4899799; doi:10.1038/srep27649)
Supplement: Supplementary Information [file srep27649-s1.pdf]

SUPPLEMENTARY INFORMATION FOR

Genetic Dissection of the Signaling Cascade that Controls Activation of the *Shigella* Type III Secretion System from the Needle Tip

Murillo[1], I. Martinez-Argudo[1,2], A. J. Blocker[3*]

[1]School of Cellular & Molecular Medicine, University of Bristol, BS8 1TD, Bristol, United Kingdom.

[2]Área de Genética, Facultad de Ciencias Ambientales y Bioquímica, Universitdad de Castilla-La Mancha, E-45071, Toledo, Spain.

[3]Schools of Cellular & Molecular Medicine and Biochemistry, University of Bristol, BS8 1TD, Bristol, United Kingdom.

*Corresponding author: ariel.blocker@bristol.ac.uk (AJB)

Supplementary Materials List

Fig. S1. Titration of arabinose concentration needed to obtain wild-type like levels of *ipaB* expression and complementation of *ipaB*$^{-}$.

Fig. S2. Quantification of Silver stained gel shown in Figure 1G.

Fig. S3. Additional characterization of properties of *ipaB** mutants.

Fig. S4. Staining patterns of antibodies used in FACS analysis of TC composition, as visualized by fluorescence microscopy.

Fig. S5. HeLa cell invasion by *ipaBxx* and *ipaBxxx* mutants.

Fig. S6. Colony color of *ipaB* and *ipaD* mutants on CR plates.

Fig. S7. Additional characterization of secretion properties of *in trans* combined *ipaB* and *ipaD* mutants and of *ipaB* mutants combined with *mxiHQ51A*.

Fig. S8. Titration of IPTG concentration needed to obtain in wild-type like levels of surface associated MxiH in *mxiH*$^{-}$/*mxiHQ51A*.

Fig. S9. Working model for conformational changes with IpaB during membrane insertion.

Supplementary Figure Legends

S1 Fig. Titration of arabinose concentration needed to obtain wild-type like levels of *ipaB* expression and complementation of *ipaB*$^-$. *ipaB*- bacteria were transformed with pBAD::*ipaB* and grown into mid-exponential phase in the presence of the indicated concentrations of arabinose. A) Exponential leakage of WT and *ipaB*$^-$*/pBAD::ipaB* were Silver stained to test complementation by recombinant IpaB. B) Quantitation of expression levels of IpaB in total culture by western blot. The relative intensity of the IpaB band was measured using Li-Cor. Results shown are representative of two independent experiments.

S2 Fig. Quantification of Silver stained gel shown in Figure 1G. Proteins Bands were quantified from the scanned gel using GeneSys. The intensities of the proteins secreted in presence of CR were normalised to WT levels and plotted.

S3 Fig. Additional characterization of properties of *ipaB mutants.** A) Exponential leakage of WT, *ipaB*$^-$, *ipaBwt* and *ipaB** mutants transformed in *ipaB*$^-$ *ipaD*$^-$ background was Silver stained (*top*) or blotted (*bottom*) to test secretability of the IpaB mutant proteins. B) Quantitation of blots for the presence of IpaB and IpaC in purified RBC membranes lysed by contact hemolysis **(Fig. 3B)**. Results shown are arithmetic means of three independent experiments; errors shown are standard deviations.

S4 Fig. Staining patterns of antibodies used in FACS analysis of TC composition, as visualized by fluorescence microscopy. Detection of IpaB, IpaD and MxiH, as compared to LPS in *Shigella* infected HeLa cell monolayers. *Left* panels, phase contrast and fluorescence overlay was used to assess the co-localisation of bacteria and antibody. Overall, all three antibodies used for FACS show discontinuous labelling around the bacterial membrane. In the *middle left* panel, phalloidin staining (*green*) was overlayed as well to show that the punctate anti-IpaB staining (*middle right*) is brighter in areas were the bacteria have initiated actin reorganization.

S5 Fig. HeLa cell invasion by *ipaBxx* and *ipaBxxx* mutants. Experiments were normalized against WT. Data represent the mean of 2 independent colonies analyzed in triplicate on the same day; error bars indicate standard deviations. Asterisks indicate statistically significant differences ($p \leq 0.05$), assessed as above.

S6 Fig. Colony color of *ipaB* and *ipaD* mutants on CR plates. Strains were plated out without and with 30 μM IPTG to overcome the possibility of white color colonies due to loss-of-function. Strains were streaked onto TCS agar supplemented with 100 μg/ml CR and left at 37°C overnight. Colony color was recorded the following day. Results shown are representative of at least two independent experiments.

S7 Fig. Additional characterization of secretion properties of *in trans* combined *ipaB* and *ipaD* mutants and of *ipaB* mutants combined with *mxiHQ51A*. A) Blots of IpaB and IpaD

levels in the supernatant of the CR induction experiment shown in **Fig. 5C**. **B)** Blots of IpaB, IpaC and IpaD levels in exponential experiment shown in **Fig. 6A, *left***.

S8 Fig. Titration of IPTG concentration needed to obtain in wild-type like levels of surface associated MxiH in *mxiH⁻/mxiHQ51A*. *mxiH⁻/mxiHQ51A* bacteria were grown into mid-exponential phase in the presence of the indicated concentrations of IPTG, surface stained for MxiH, IpaD and IpaB and analyzed by FACS. *mxiH⁻* (*H⁻*) is used as a negative control, wild-type (WT), *ipaB- mxiH-/ipaBwt mxiHQ51A* (*BHQ51A*) and *ipaD- mxiH-/ipaDwt mxiHQ51A* (*DHQ51A*) are used as positive controls with 20 μM IPTG. Data is expressed as percent brightness of WT.

S9 Fig. Working model for conformational changes with IpaB during membrane insertion. *Left*, IpaB (different linear domains shown with same color scheme as in **Fig. 1A**) atop a needle (MxiH protofilaments shown in thick purple lines), next to two IpaD subunits (thin purple lines), prior to host cell contact. Asterisk indicates hypothetical location of the surface-accessible epitope of anti-IpaB mAb antibody H16, which is known to recognize the tip of the alacoil [1,2]. *Right*, hypothetical conformation of membrane inserted IpaB. Note the similarity in amino acid (AA) length of the portions of IpaB on either side of its membrane-associated section, N-terminal domain excluded. These long sections are proposed to form a long coiled-coil.

References

1 Veenendaal, A. K. *et al.* The type III secretion system needle tip complex mediates host cell sensing and translocon insertion. *Mol Microbiol* **63**, 1719-1730, doi:MMI5620 [pii]
10.1111/j.1365-2958.2007.05620.x (2007).

2 Shen, D. K., Saurya, S., Wagner, C., Nishioka, H. & Blocker, A. J. Domains of the Shigella flexneri type III secretion system IpaB protein involved in secretion regulation. *Infect Immun* **78**, 4999-5010, doi:IAI.00470-10 [pii]
10.1128/IAI.00470-10 (2010).

Figure S1

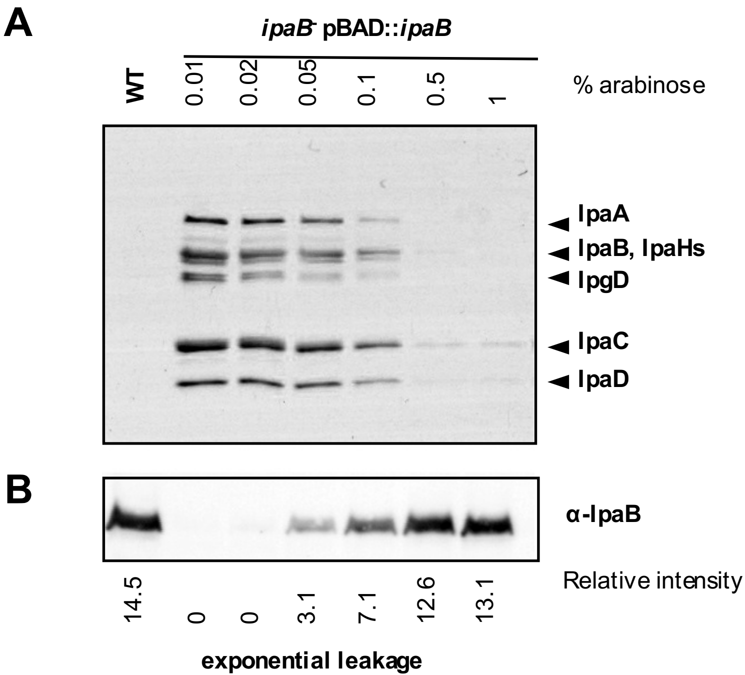
A
ipaB- pBAD::ipaB
WT
0.01
0.02
0.05
0.1
0.5
1
% arabinose
IpaA
IpaB, IpaHs
IpgD
IpaC
IpaD
B
α-IpaB
Relative intensity
14.5
0
0
3.1
7.1
12.6
13.1
exponential leakage

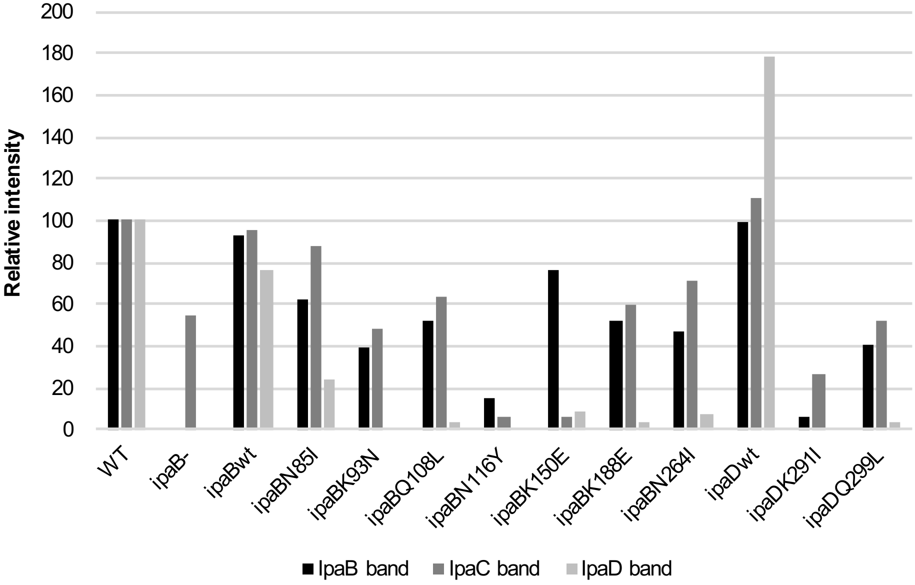
200
180
160
140
120
100
80
60
40
20
0
Relative intensity
WT
ipaB-
ipaBwt
ipaBN85I
ipaBK93N
ipaBQ108L
ipaBN116Y
ipaBK150E
ipaBK188E
ipaBN264I
ipaDwt
ipaDK291I
ipaDQ299L
IpaB band
IpaC band
IpaD band

Figure S2

Figure S3

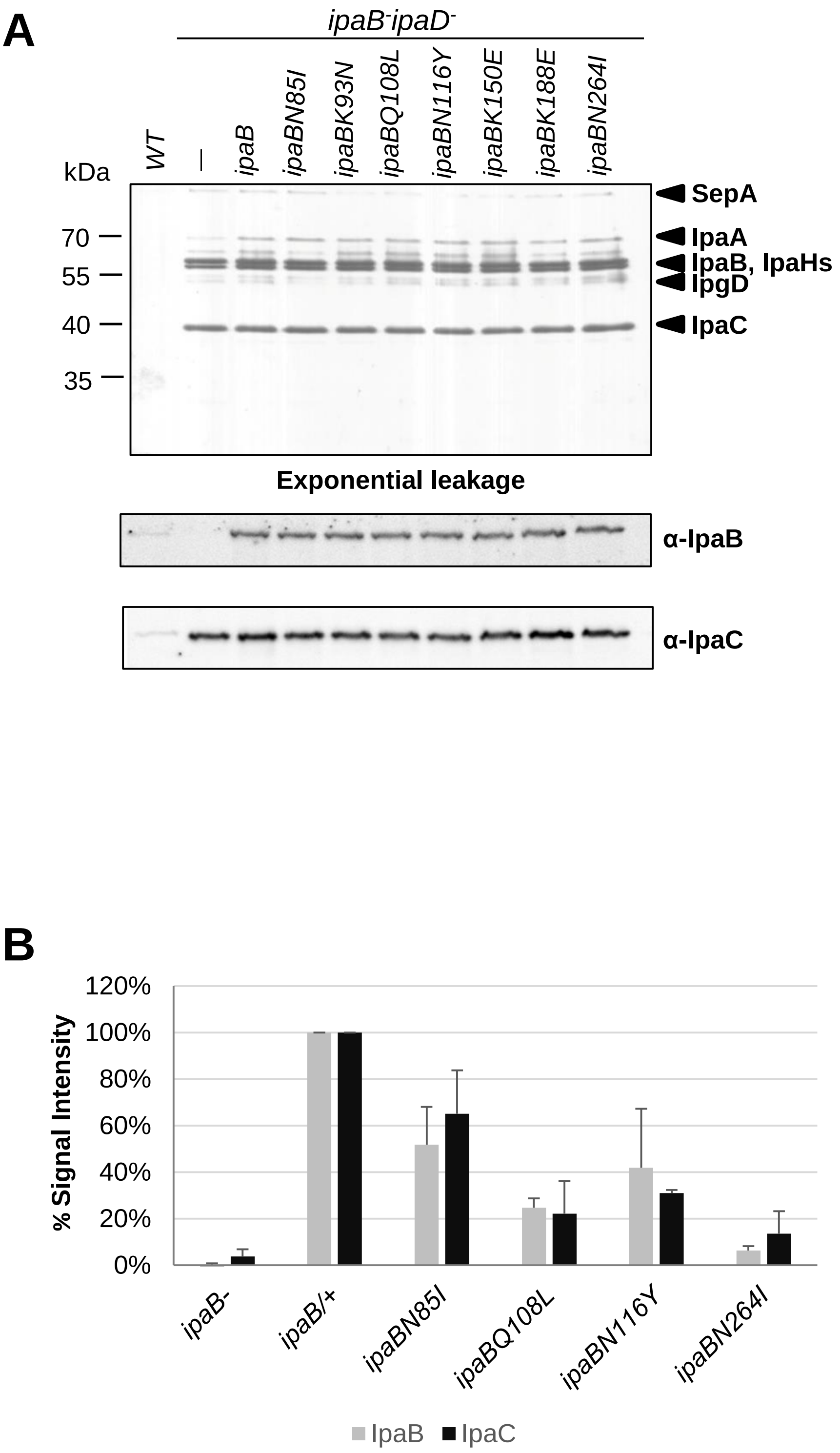
A
ipaB⁻ipaD⁻
WT
—
ipaB
ipaBN85I
ipaBK93N
ipaBQ108L
ipaBN116Y
ipaBK150E
ipaBK188E
ipaBN264I
kDa
70
55
40
35
SepA
IpaA
IpaB, IpaHs
IpgD
IpaC
Exponential leakage
α-IpaB
α-IpaC
B
% Signal Intensity
120%
100%
80%
60%
40%
20%
0%
ipaB-
ipaB/+
ipaBN85I
ipaBQ108L
ipaBN116Y
ipaBN264I
IpaB
IpaC

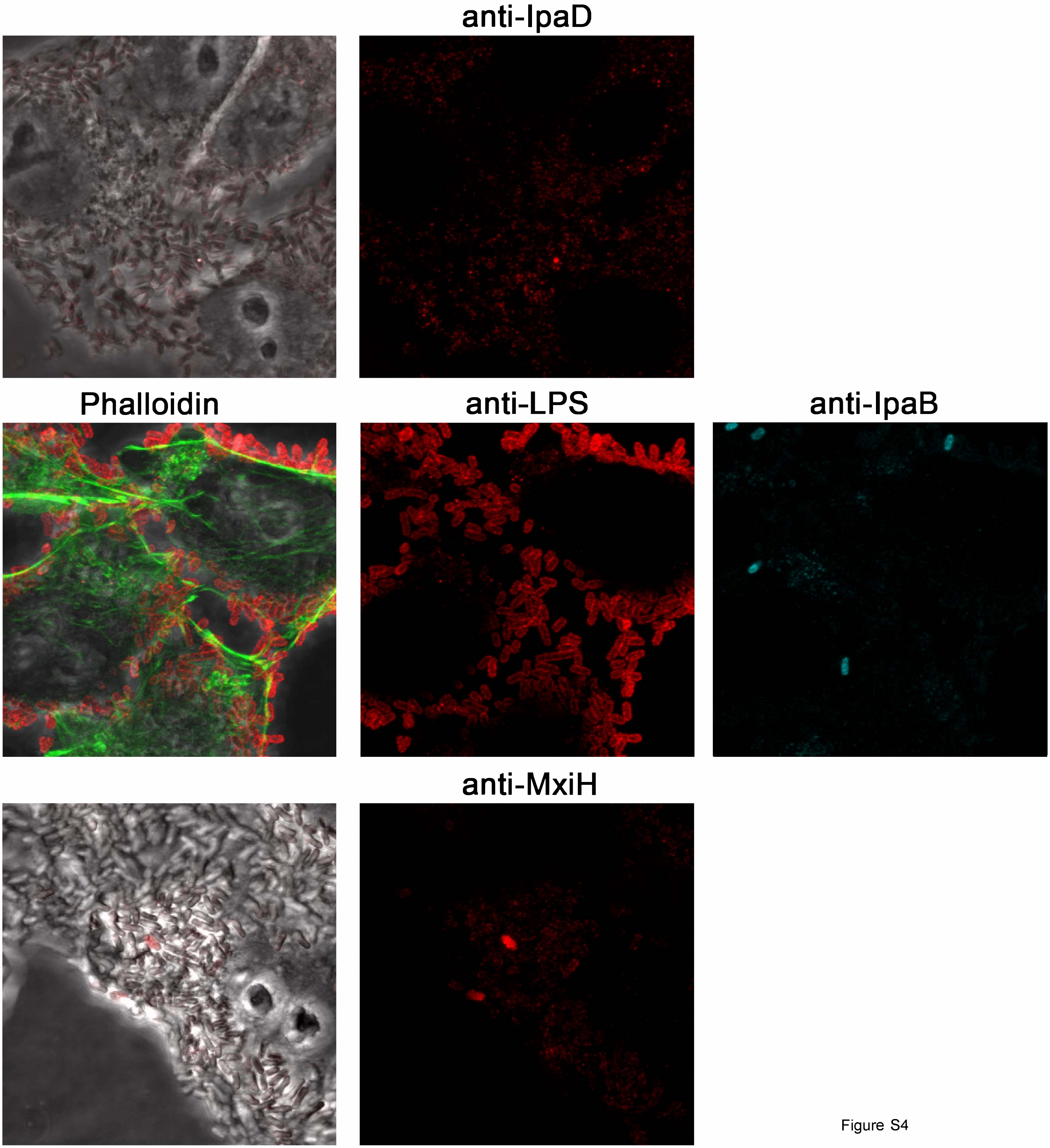

Figure S4

Figure S5

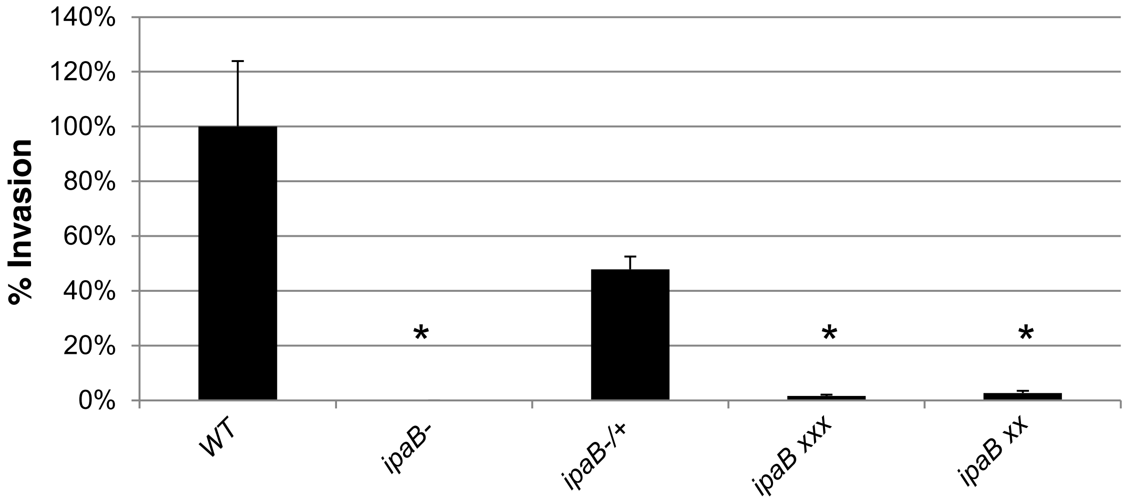

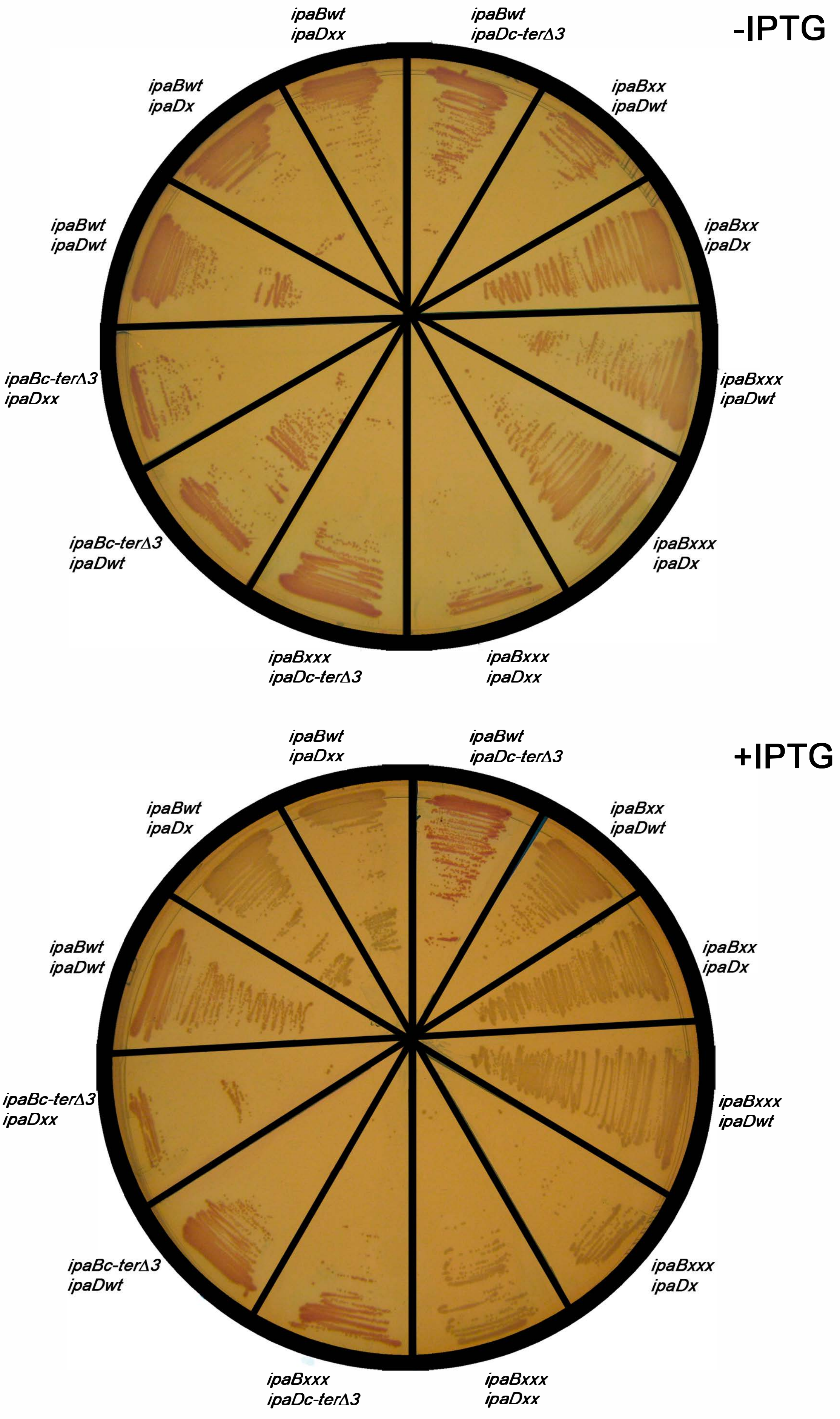
-IPTG
ipaBwt
ipaDxx
ipaBwt
ipaDc-terΔ3
ipaBxx
ipaDwt
ipaBxx
ipaDx
ipaBxxx
ipaDwt
ipaBxxx
ipaDx
ipaBxxx
ipaDxx
ipaBxxx
ipaDc-terΔ3
ipaBc-terΔ3
ipaDwt
ipaBc-terΔ3
ipaDxx
ipaBwt
ipaDwt
ipaBwt
ipaDx
+IPTG
ipaBwt
ipaDxx
ipaBwt
ipaDc-terΔ3
ipaBxx
ipaDwt
ipaBxx
ipaDx
ipaBxxx
ipaDwt
ipaBxxx
ipaDx
ipaBxxx
ipaDxx
ipaBxxx
ipaDc-terΔ3
ipaBc-terΔ3
ipaDwt
ipaBc-terΔ3
ipaDxx
ipaBwt
ipaDwt
ipaBwt
ipaDx

Figure S7

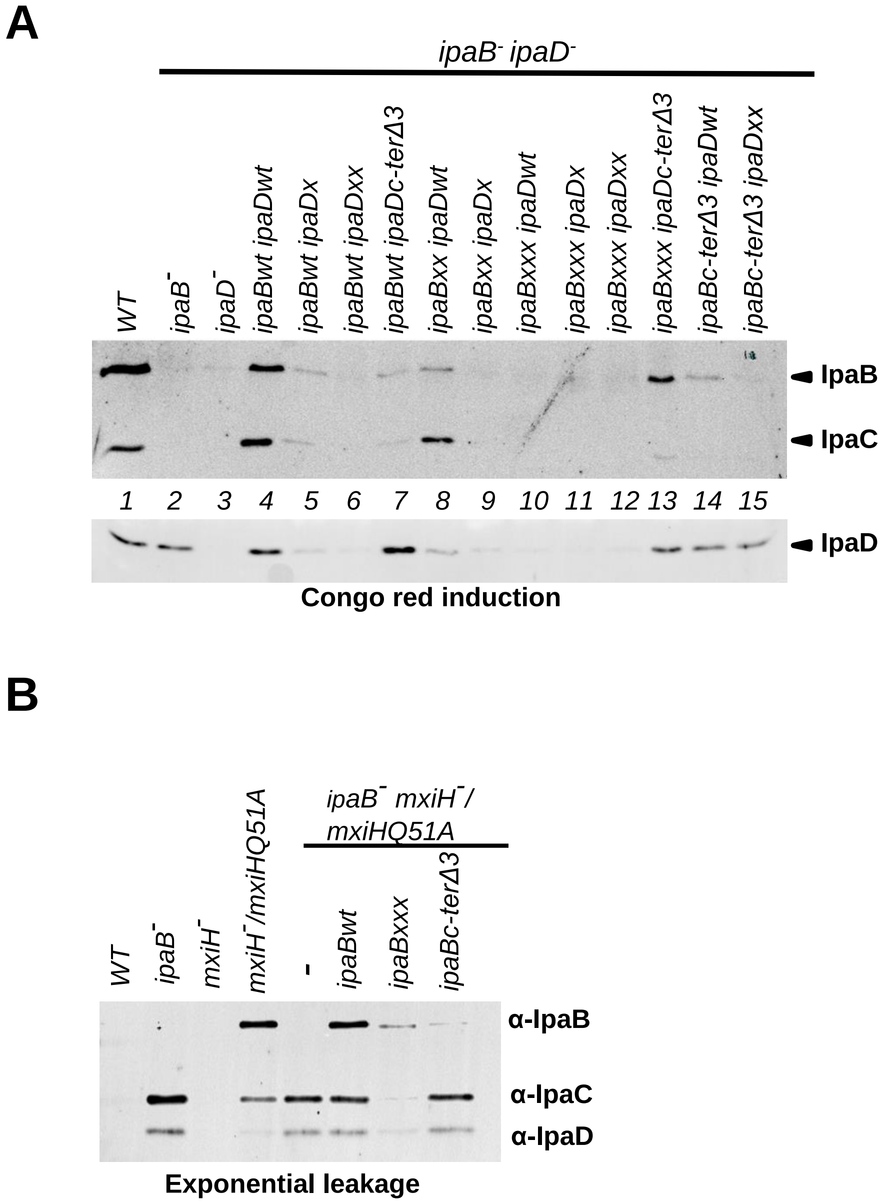
A
ipaB- ipaD-
WT
ipaB-
ipaD-
ipaBwt ipaDwt
ipaBwt ipaDx
ipaBwt ipaDxx
ipaBwt ipaDc-terΔ3
ipaBxx ipaDwt
ipaBxx ipaDx
ipaBxxx ipaDwt
ipaBxxx ipaDx
ipaBxxx ipaDxx
ipaBxxx ipaDc-terΔ3
ipaBc-terΔ3 ipaDwt
ipaBc-terΔ3 ipaDxx
IpaB
IpaC
1 2 3 4 5 6 7 8 9 10 11 12 13 14 15
IpaD
Congo red induction
B
ipaB- mxiH-/ mxiHQ51A
WT
ipaB-
mxiH-
mxiH-/mxiHQ51A
-
ipaBwt
ipaBxxx
ipaBc-terΔ3
α-IpaB
α-IpaC
α-IpaD
Exponential leakage

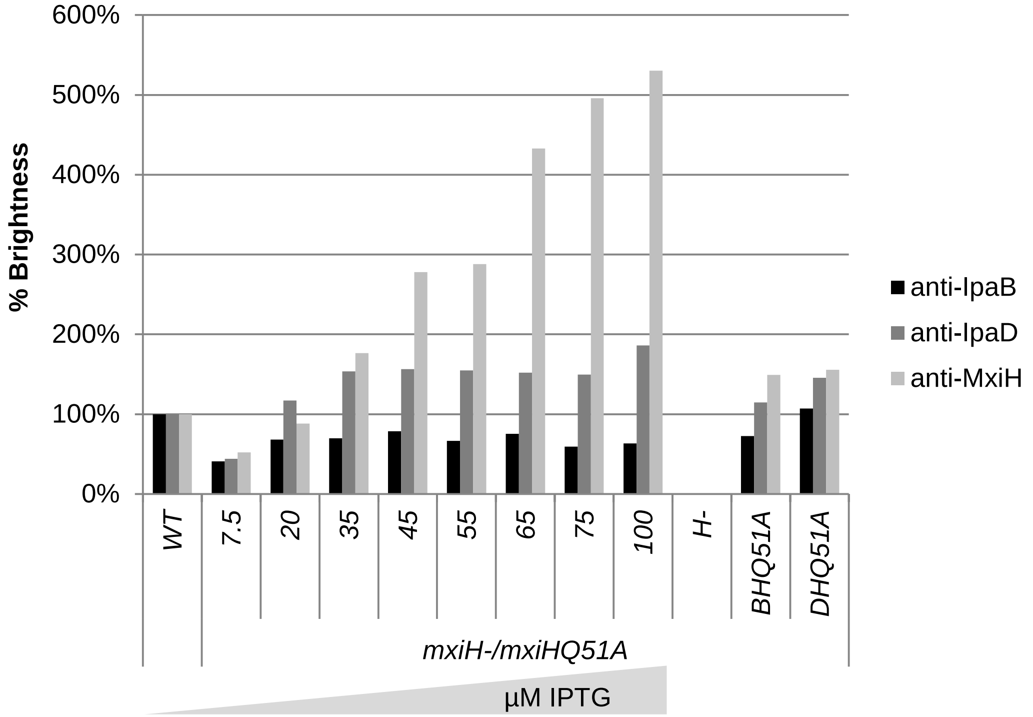
% Brightness
600%
500%
400%
300%
200%
100%
0%
anti-IpaB
anti-IpaD
anti-MxiH
WT
7.5
20
35
45
55
65
75
100
H-
BHQ51A
DHQ51A
mxiH-/mxiHQ51A
µM IPTG

Figure S9

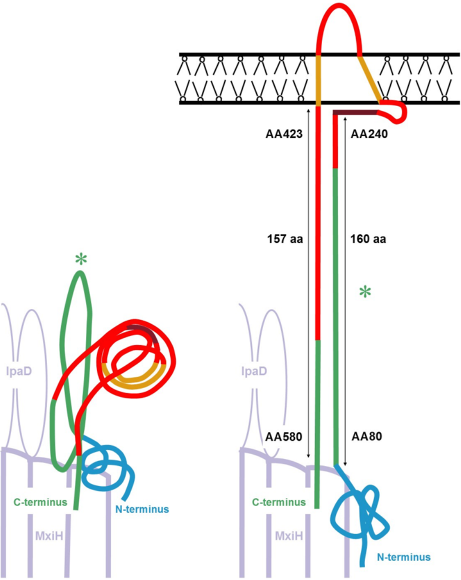
AA423
AA240
157 aa
160 aa
IpaD
AA580
AA80
C-terminus
N-terminus
MxiH
IpaD
C-terminus
MxiH
N-terminus
